# Efficacy in deceptive vocal exaggeration of human body size

**DOI:** 10.1038/s41467-021-21008-7

**Published:** 2021-02-12

**Authors:** Katarzyna Pisanski, David Reby

**Affiliations:** 1 Equipe de Neuro-Ethologie Sensorielle (ENES), Centre de Recherche en Neurosciences de Lyon (CRNL), CNRS, INSERM, University of Lyon/Saint-Étienne, Saint-Étienne, France; 2 Institute of Psychology, University of Wrocław, Wrocław, Poland

**Keywords:** Sexual selection, Human behaviour, Animal behaviour

## Abstract

How can deceptive communication signals exist in an evolutionarily stable signalling system? To resolve this age-old honest signalling paradox, researchers must first establish whether deception benefits deceivers. However, while vocal exaggeration is widespread in the animal kingdom and assumably adaptive, its effectiveness in biasing listeners has not been established. Here, we show that human listeners can detect deceptive vocal signals produced by vocalisers who volitionally shift their voice frequencies to exaggerate or attenuate their perceived size. Listeners can also judge the relative heights of cheaters, whose deceptive signals retain reliable acoustic cues to interindividual height. Importantly, although vocal deception biases listeners’ absolute height judgments, listeners recalibrate their height assessments for vocalisers they correctly and concurrently identify as deceptive, particularly men judging men. Thus, while size exaggeration can fool listeners, benefiting the deceiver, its detection can reduce bias and mitigate costs for listeners, underscoring an unremitting arms-race between signallers and receivers in animal communication.

## Abstract (web summary)

While size exaggeration is common in the animal kingdom, Pisanski & Reby show that human listeners can detect deceptive vocal signals of people trying to sound bigger or smaller, and recalibrate their estimates accordingly, especially men judging the heights of other men, with implications for the evolution of vocal communication.

## Introduction

The honest signalling paradox has perplexed researchers in animal communication for five decades^1–9^. Indeed, why should receivers pay attention to deceptive signals if these signals contain little to no reliable information? Conversely, if receivers detect and correct for deception, why should signallers continue to produce potentially costly deceptive displays?

At the crux of this paradox lies the inherent conflict of interest between signallers and receivers. While earlier theories saw animal communication as a cooperative exchange of information^10^, the production of animal signals during communication between unrelated individuals is now predominantly regarded as a selfish behaviour^2,10^, whereby signallers attempt to manipulate the responses of receivers. Crucially, however, selection also operates on receivers to evade deception, for example by leading them to ignore deceptive signals or to recalibrate their responses. Indeed both individuals in a dyadic exchange are expected to behave in ways that maximise their own fitness^2^, giving rise to an evolutionary arms race that is most apparent when interests diverge (e.g., mate choice) or are entirely opposed (e.g., resource contests)^11^. Yet, even in ostensibly cooperative contexts such as alarm calling^12^, signallers may stand to gain substantial fitness benefits by exaggerating (or in some cases, attenuating^13^) signals, if such deceptive signals succeed to elicit a beneficial response from receivers^6,7^.

To remain ‘evolutionarily stable’^14^, signals must confer net fitness benefits to both senders and receivers^2^, and thus should be generally reliable or ‘honest on average’^4,15^. Even a putatively deceptive signal should be reliable enough to remain beneficial for receivers to attend to it. In other words, not only can deception and reliability coexist, deception depends on reliability, because without some element of truth a signalling system would collapse^6^. Signal reliability can be imposed by a number of mechanisms, including anatomical or physiological constraints (e.g., by-product information^16^ or honest indices^15^), developmental or metabolic costs^1,17^, and reputation or retaliation costs^9,18^. Constraints and costs, if high enough, can enforce signal honesty. Nevertheless, even a presumably reliable signal will be ‘incompletely honest’^7^ due either to deceptive processes that must operate within these constraints, including anatomical adaptations to the vocal apparatus in many mammals^19^, or nondeceptive processes, including developmental noise^20^, communication errors, or environmental signal degradation^7^. While challenging^6^, it is imperative to dissociate deceptive from nondeceptive processes that can independently degrade signal reliability if we are to truly understand the evolution of a signalling system^7^.

Signal reliability is often measured as the strength of the correlation between the signal and the intended information^6,7^. To gauge signal reliability we must establish (a) if the signal is reliable enough that a receiver will generally benefit by attending to it; (b) the constraints or costs that impose this degree of honesty; and perhaps most critically, (c) how receivers respond to the signal, including detecting and compensating, at least in part, for any deception^6^. Two major obstacles have limited the extent to which we can answer these questions using animal models: uncertainty in what a signal is actually intended to convey and uncertainty in what a receiver is attending to^6^.

**Fig. 1:**
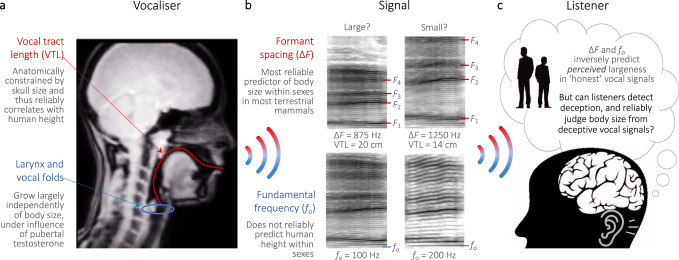
Human vocal communication of body size. **a** Sagittal MRI image of human vocal anatomy illustrating vocal tract length (VTL) (in red) during production of the vowel /u:/, and permanently descended larynx housing the vocal folds (blue circle). Human VTL scales fairly allometrically with body size due to anatomical constraints, whereas vocal fold length does not^26,28,32,33^. **b** Resonances of the vocal tract termed formants (labelled *F*_1_–*F*_4_) and their relative spacing (∆*F*) inversely predict human height controlling for sex and age^33,34^, whereas fundamental frequency (labelled *f*_o_), related to vocal fold length, does not^34^. **c** Human listeners robustly associate both voice frequencies (low ∆*F* and *f*_o_) with large body size in regular speech^25,36^ and in nonverbal vocalisations (e.g., roars^39^), but can humans judge size from deceptive vocal signals? Panels **a, b** were reproduced and reformatted with permission^21^; parts of panel **c** were designed using resources from freepik.com and rawpixel.com (https://www.freepik.com/free-vector/illustration-business-people_2609966.htm#query=man%20black%20silhouette&position=17).

We propose that studying deception in human communication signals offers a promising solution to these long-standing hurdles. Unlike other animals, humans can produce specific deceptive signals on demand^9,21–23^, thus eliminating uncertainty in the signal’s intended function and allowing researchers to pin-point the contribution of deception in signal reliability. Moreover, researchers can directly measure the effects of this deception on human receivers using controlled psychoacoustic experiments, offering a full picture of the signaller–receiver communication chain (Fig. 1). Here, we apply this paradigm to study an ecologically relevant vocal signalling system observed across the animal kingdom—vocal communication of body size.

Given the often substantial fitness benefits of a large body size, especially for males who gain access to resources and mates^11,24^ (tall men included^25^), it is unsurprising that many species of mammals, birds, fish, reptiles, amphibians, and arthropods have evolved anatomical or behavioural adaptations to exaggerate their apparent size^6,19,26–28^. For example, the already-descended larynx of red deer stags^29^ is lowered further still during roaring contests with rival males^30^, extending the vocal tract even more to produce abnormally low formant frequency spacing (∆*F*, the overall spacing between any two consecutive formants in the frequency domain, see Fig. 1) given the animal’s true size^27,31^.

Humans also possess a descended and sexually dimorphic larynx, with men boasting longer vocal tracts (reduced ∆*F*) and longer vocal folds (lower fundamental frequency or pitch, *f*_o_) than women^28,32,33^ (Fig. 1). Although ∆*F* scales allometrically with vocal tract length (VTL) and thus predicts body size, both between and within adult sexes, *f*_o_ is a poor predictor of human height at the intrasexual level^28,33,34^ (see Fig. 1). Yet, despite strongly associating not only ∆*F* but also *f*_o_ with physical largeness^25,35,36^, listeners can gauge relative body size from modal speech and nonverbal vocalisations^25,36,37^. Critically, however, while we have recently shown that men and women can behaviourally lower their voice ∆*F* and *f*_o_ to further exaggerate their body size and strength^38,39^, remarkably little is known about the role of such deception in size communication.

In this study, we combine acoustic analysis of vocal signals, produced by men and women attempting to sound physically larger or smaller, with a series of psychoacoustic playback experiments conducted on a representative sample of 200 human listeners. Their task was to judge the heights of these vocalisers, and to attempt to discriminate among honest, exaggerated, or attenuated vocal signals of body size. Using this innovative approach we address long-standing questions about the evolution of deceptive signals in animal communication: Does deceptive size signalling retain an element of honesty? Can listeners detect size deception and do they correct for it when judging height? If so, does it still benefit vocalisers to exaggerate their perceived size? Are there sex differences, as predicted by sexual selection^11,25^, in the production and perception of exaggerated signals? Our results offer a unique lens into the conflict between deceivers and receivers, showing that while deceptive vocal signals can effectively bias listeners’ judgements of body size, such signals remain constrained and thus retain some reliable information. Specifically, we show that listeners often correctly discriminate between honest and deceptive vocal signals, and that when they do detect deception, they can recalibrate their height judgements accordingly. This research reveals that although listeners are not systematically fooled, it still pays off to deceive.

## Results

### Voice frequency shifts in size deception

As a first step toward answering our key research questions, we analysed speech signals (vowels /α i ɛ o u/) produced by men and women tasked with sounding physically larger and smaller than their true body size^38^. Acoustic analyses performed in Praat^40^ (see Methods) confirmed that both sexes volitionally lowered their voice fundamental frequency (*f*_o_) and formant spacing (∆*F*) to deceptively exaggerate their apparent body size, and raised both frequency parameters to attenuate it, relative to their unmodulated (herein ‘honest’) vocal signals.

Men, by extending their apparent VTL more extremely than did women, lowered their formants more (Supplementary Tables 1 and 2), which is expected to simulate a larger body size (Fig. 1). In contrast, men did not raise their formants significantly more than did women to sound smaller, nor did they shift their voice pitch (*f*_o_) more (LMM, Supplementary Table 2). The observed sex difference in formant shifts cannot be attributed to sexual dimorphism in starting VTL as controlling for baseline VTL or ∆*F* showed that the mean percentage change in men’s formant shifts was still three to four times greater than that of women (Supplementary Table 1). Even within sexes, taller men likewise shifted their formants during size deception more than did shorter men, wherein relative differences in men’s heights explained one-third of the variance in formant shift magnitude for size exaggeration. Again, this relationship was not observed among women, nor for *f*_o_ shifts in either sex (Supplementary Table 3). Taken together, these results support the hypothesis that the human male vocal tract may have evolved largely under selection pressure for size exaggeration^21,28^, wherein men may perform vocal tract dynamics to maximise their perceived body size, and ultimately, their reproductive success^25^, while simultaneously preserving phonetic range as illustrated by articulatory models^41^.

Formant measures (∆*F* and apparent VTL) taken from central vowel frequencies in honest vocal signals explained between 14% and 40% of the variance in actual height within sexes, whereas *f*_o_ explained virtually none (<3%; Supplementary Table 4). These findings corroborate studies on several other terrestrial mammals^26,27^ including humans (see the meta-analysis in ref. ^34^), where formants but not *f*_o_ are anatomically constrained and thus follow a degree of acoustic allometry (Fig. 1). Importantly, formant measures also predicted inter-individual differences in height from deceptive vocal signals, particularly among men exaggerating their size (∆*F R*^2^ = 0.58) and women attenuating their size (∆*F R*^2^ = 0.17; Supplementary Table 4). Here too, *f*_o_ did not significantly predict individual differences in actual height from deceptive signals (*R*^2^ = 0.003–0.12; Supplementary Table 4). These acoustic analyses show that reliable formant-based information indicating inter-individual differences in body size is present in the human voice even during size deception, and thus, that listeners may be able to reliably gauge relative size from deceptive vocal signals.

### Vocal size deception biases listeners

Do these deceptive signals fool listeners? To answer this imperative question, human adults (Experiment 1: *n* = 97, aged 18–63, 59 males) completed two psychoacoustic tasks involving (1) judging the absolute heights of vocalisers from their honest and deceptive vocal signals using a sliding metric/imperial scale, and (2) judging whether those same vocalisers were speaking naturally or instead deceptively exaggerating or attenuating their size (see Methods). To avoid cueing listeners to the possibility of vocal deception and thus to maintain ecological validity, listeners judged the height before assessing deception in a separate experimental block (but see Experiment 2).

**Fig. 2:**
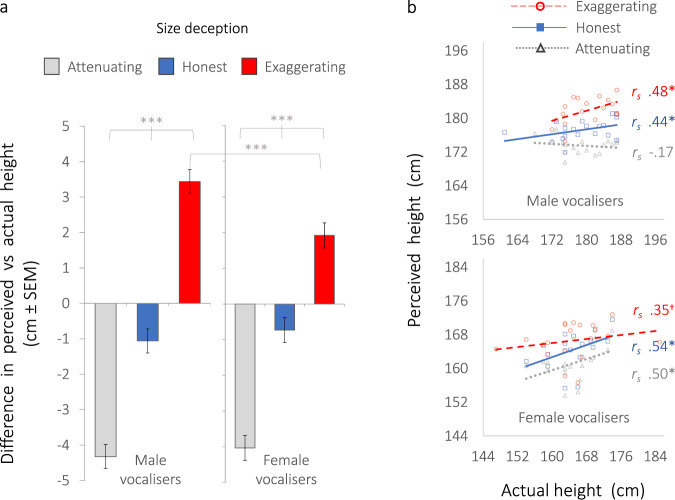
Vocal size deception biases judgements of body size (Experiment 1). **a** Bias in height judgements shown as the mean difference (±SEM) between perceived and actual heights of vocalisers, in cm, for honest vocal signals (central blue bars) and deceptive vocal signals (attenuating size = grey bars, exaggerating size = red bars), where 0 indicates accurate height judgements, positive values indicate overestimation, and negative values indicate underestimation. Estimated marginal means and pairwise comparisons derive from linear mixed models, LMMs (see Supplementary Table 5), where all ***p < 0.001 following Šidák correction for multiple comparisons. Tests are two-tailed. Error bars represent standard errors of the mean, ±SEM. See also Supplementary Fig. 1 for dot plots illustrating the distribution of data. **b** Linear regressions showing relationships between perceived and actual heights of vocalisers, where each dot represents a vocaliser, and height judgements are averaged across listeners for each vocaliser and each size condition (exaggerating size = red circles, honest = blue squares, attenuating = grey triangles), where Cook’s Di < 0.20 (see Methods). 1 cm on the *x*-axis is equal to 1 cm on the *y*-axis. Spearman’s *rho* (*r*_s_) correlation coefficients are given for each regression line, where *p < 0.05, one-tailed (males exaggerating *r* = 0.48, *p* = 0.02, *n* = 18; honest *r* = 0.44, *p* = 0.03, *n* = 19; attenuating *r* = −0.17, *p* = 0.25, *n* = 18; females exaggerating *r* = 0.35, *p* = 0.06, *n* = 20; honest *r* = 0.54, *p* = 0.01; *n* = 18; attenuating *r* = 0.50, *p* = 0.02; *n* = 18). All data were de*r*ived from Experiment 1 based on 120 vocal stimuli produced by *n* = 40 vocalisers (20 males, 20 females) in each of three size conditions (honest, attenuating, exaggerating) and judged by *n* = 97 listeners, where each vocal stimulus was rated by an average of 50 listeners (see Methods). Source data are provided as a Source Data file.

Linear mixed models (LMMs) confirmed that listeners’ height judgements were indeed biased by size deception when judging both male (*F*_2,1938_ = 299.2, *p* < 0.001) and female vocalisers (*F*_2,1938_ = 174.3 *p* < 0.001), with no effects of listener sex (Supplementary Table 5). As illustrated in Fig. 2a, on average, listeners overestimated the height of size exaggerators (estimated marginal means, *M* 3.4 cm, 95% CI 2.8, 4.1 male voices; *M* 1.9 cm, 95% CI 1.23, 2.6 female voices) and underestimated the height of size attenuators, by an average of 4 cm (*M* −4.3 cm, 95% CI −5.0, −3.7 male voices; *M* −4.0 cm, 95% CI −4.7, −3.3 female voices). Men attempting to exaggerate (but not attenuate) their size biased listeners’ judgements more effectively than did women (Fig. 2a; *F*_2,3872_ = 9.9, *p* < 0.001; Supplementary Table 5), consistent with previous suggestions that the exaggeration of apparent body size is under stronger sexual selection in male than female vocal signals^11,25,26,35^, especially in sexually dimorphic species in which males are larger than females^19^.

The moderate positive relationship between actual and perceived height in men’s honest vocal signals (*r* = 0.44) was retained in their exaggerated (*r* = 0.48) but not attenuated signals (Fig. 2b). In women, honest (*r* = 0.54) and attenuated (*r* = 0.50) signals showed the strongest relationships (Fig. 2b). Thus, although listeners’ individual height judgements were systematically shifted up by size exaggeration and down by size attenuation, inter-individual differences in height were preserved in listeners’ judgements. This could be expected based on our acoustic analyses indicating reliable formant-based cues to actual size in deceptive vocal signals (Supplementary Table 4). As further predicted, listeners generally associated lower absolute voice frequencies with larger size, a well-established perceptual association^25,27,36,37^. However, this association was stronger for exaggerated than honest or attenuated signals (Supplementary Table 6). Together these results suggest that lowering voice frequencies can function to effectively maximise perceived absolute body size, again to the potential fitness benefit of the size exaggerator^11,25,35^.

### Listeners can detect deception

**Fig. 3:**
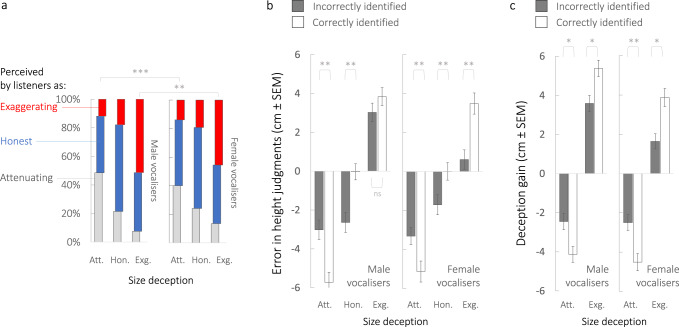
Listeners can detect deception, but remain deceived by deceptive signals (Experiment 1). **a** Percentages of vocalisers that listeners perceived as deceptively exaggerating (red bars) or attenuating (light grey bars) their size, or as producing honest vocal signals (blue bars, centre) are shown along the *y*-axis as a function of the intended size deception indicated along the *x*-axis. Estimated marginal means and pairwise comparisons derive from LMMs (see Supplementary Table 7), where ***p < 0.001, **p < 0.01, following Šidák correction for multiple comparisons. Tests are two-tailed. **b**, **c** Bias in listeners’ size assessments as a function of whether a listener failed to detect (dark grey bars) or correctly detected (white bars) a vocal signal as deceptive or honest, where panel **b** shows ‘error’ in height judgements (mean difference between perceived vs actual heights of vocalisers), and panel **c** shows ‘deception gain’ in height judgements (mean difference between perceived height from honest signals and perceived height from deceptive signals). Estimated marginal means and pairwise comparisons derive from LMMs (see Supplementary Tables 8 and 9), **p < 0.01, *p < 0.05 following Šidák correction. Tests are two-tailed. Error bars ± SEM. Acronyms: Att. attenuating, Hon. honest, Exg. exaggerating. All data derive from Experiment 1, based on 120 vocal stimuli produced by *n* = 40 vocalisers (20 males, 20 females) in each of three size conditions (honest, attenuating, exaggerating) and judged by *n* = 97 listeners, where each vocal stimulus was rated by an average of 50 listeners (see Methods). See also Supplementary Fig. 2 for dot plots illustrating distribution of data. Source data are provided as a Source Data file.

In a second task, the same listeners judging the same vocal stimuli correctly detected the presence or absence of size deception above chance (Fig. 3a; chance = 33%, grand mean correct 51% ± 0.007 SEM, 95% CI 49–52%) and more reliably detected deception by men than deception by women during intended size exaggeration (51% ± 0.02 vs 45% ± 0.02) and attenuation (49% ± 0.02 vs 40% ± 0.02) with no differences between male and female listeners (LMM, Supplementary Table 7, Fig. 3a).

A crucial question then is whether listeners recalibrated their height judgements for voice signals that they were able to correctly detect as deceptive in a subsequent task. As illustrated in Fig. 3b (LMMs, Supplementary Tables 8 and 9), honest signals correctly identified as such were indeed associated with reliable height judgements overall (i.e., error near 0, *M* −0.035 cm, 95% CI −0,8, −0.78 male voices; *M* 0.009 cm, 95% CI −0.87, 0.89 female voices), whereas honest signals misidentified as deceptive were underestimated (*M* −2.6 cm, 95% CI −3.6, −1.6 male voices; *M* −1.7 cm, 95% CI −2.7, −0.7 female voices). Surprisingly, however, listeners’ judgements of height were maximally biased for deceptive signals they correctly identified as deceptive in a subsequent task (Fig. 3b, c).

These results indicate that listeners remain partly ‘fooled’ by vocal deception, and were in fact most fooled by voices they could correctly identify as deceptive in a separate task. We examined the possibility that detectable deceivers shifted their voice frequencies more than those who remained undetected, which could increase detection while also maximally exploiting deep-seated sound-size correspondences between low frequencies and largeness^25,27,36,37^. Although women who were detected as exaggerating did indeed shift their ∆*F* and *f*_o_ more than those who remained undetected (Supplementary Table 10), such acoustic differences were not observed among men nor in the context of size attenuation. Hence, we tested a second possibility that the listeners in this experiment, who were not ‘primed’ to contemplate deception when judging the heights of vocalisers, may have failed to detect deception at that stage, and thus to recalibrate their judgements.

### Awareness of deception reduces bias

**Fig. 4:**
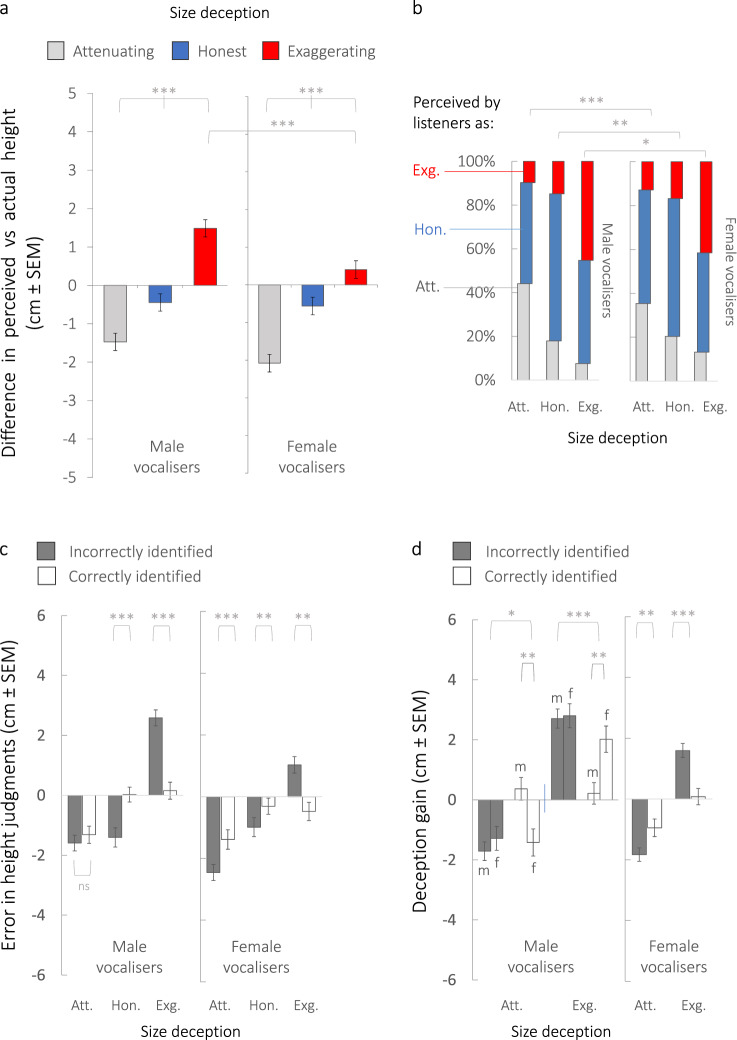
Awareness reduces bias: Listeners recalibrate height judgements for signals correctly and concurrently detected as deceptive (Experiment 2). **a** Bias in height judgements shown as the mean difference (±SEM) between perceived and actual heights of vocalisers, in cm, for honest vocal signals (blue bars) and deceptive vocal signals (attenuating = grey bars, exaggerating = red bars). Estimated marginal means and pairwise comparisons derive from LMMs (see Supplementary Table 11), where all ***p < 0.001 following Šidák correction for multiple comparisons. Error bars ±SEM. **b** Percentages of vocalisers that listeners perceived as deceptively exaggerating (red bars) or attenuating (light grey bars) their size, or as producing honest vocal signals (blue bars, centre) are shown along the *y*-axis as a function of the intended size deception indicated along the *x*-axis. Estimated marginal means and pairwise comparisons derive from LMMs (see Supplementary Table 12), where ***p < 0.001, **p < 0.01, *p < 0.05 following Šidák correction. Tests are two-tailed. **c**, **d** Bias in listeners’ size assessments as a function of whether a listener failed to detect (dark grey bars) or correctly detected (white bars) a vocal signal as deceptive or honest, where panel **c** shows ‘error’ in height judgements (mean difference between perceived and actual heights of vocalisers), and panel **d** shows ‘deception gain’ in height judgements (mean difference between perceived height from honest signals and perceived height from deceptive signals). Panel **d** also illustrates that deception gain was lower for male vocalisers (left side) when correctly detected as cheating by other male listeners (labelled with ‘m’) compared to when detected by female listeners (‘f’). Estimated marginal means and pairwise comparisons derive from LMMs (see Supplementary Tables 13 and 14), ***p < 0.001, **p < 0.01, *p < 0.05 following Šidák correction. Tests are two-tailed. Error bars ± SEM. Acronyms: Att. attenuating, Hon. honest, Exg. exaggerating, m male listeners, f female listeners. All data derive from Experiment 2 based on 120 vocal stimuli produced by *n* = 40 vocalisers (20 males, 20 females) in each of three size conditions (honest, attenuating, exaggerating) and judged by *n* = 98 listeners, each of whom rated all 120 vocal stimuli (see Methods). See also Supplementary Fig. 3 for dot plots illustrating the distribution of data. Source data are provided as a Source Data file.

To test this possibility, we conducted a second psychoacoustic experiment on an independent sample of listeners (Experiment 2: *n* = 98, aged 18–71, 59 males). The experiment was identical to the first, except that listeners now completed both tasks concurrently, first indicating whether or not they perceived a vocaliser as deceptively altering their size, and then judging the height of that same vocaliser within the same trial (see Methods). The results confirmed our prediction (Fig. 4; LMMs, Supplementary Tables 11–14). Indeed, listeners primed to seek deceit were substantially less biased by vocal size exaggeration, overestimating or underestimating the heights of vocal deceivers by half the magnitude as listeners in Experiment 1 (Fig. 4a vs Fig. 2a and Supplementary Table 11). Although listeners in both experiments detected size deception with similar verity (Fig. 4b vs Fig. 3a and Supplementary Table 12), critically, listeners in Experiment 2 more effectively recalibrated their height judgements for vocal signals they correctly identified as deceptive (Fig. 4c, d vs Fig. 3b, c and Supplementary Tables 13, 14). This effect was most pronounced for size exaggerators who, when correctly detected as cheating, failed to fool listeners into perceiving them as much larger than their true body size (Fig. 4c, d). Moreover, male listeners recalibrated their size judgements after correctly detecting deception more effectively than did female listeners, specifically when assessing the body size of other men (Fig. 4d left panel and LMM, Supplementary Table 14).

## Discussion

Our results provide rare insight into an evolutionary arms race between signallers and receivers, addressing several long-standing questions aimed at resolving the honest signalling paradox^1–9^. First, is honesty retained in deceptive signals? Yes. Presumably due to anatomical constraints on VTL^26,28^ limiting the extent of deception, the relationship between formant frequencies and true body size (acoustic allometry) is preserved, albeit shifted, during vocal size deception. The resulting reliable cues to intra-individual differences in height ostensibly allowed listeners in the present study to ‘rank’ the relative heights of cheating vocalisers. Second, can listeners detect deception? Yes. Listeners correctly identified vocal signals as honest or deceptive much more often than expected by chance, though they nevertheless failed to detect deception approximately half of the time. Third, do listeners recalibrate their height judgements when they detect deception? Yes, largely, and much more if they are primed to seek deception. Indeed in Experiment 2, height judgements were significantly less biased for vocal signals correctly and concurrently detected as deceptive. Fourth, and crucially, does it still pay to deceive? Yes. We show that vocalisers who attempted to alter their apparent body size by shifting the frequency parameters of their voice effectively fooled listeners, who indeed perceived them as taller or shorter, by several centimetres on average. Although primed detection of deception reduced this bias, listeners’ height judgements remained biased overall, as deceit often went undetected.

Our results also show that males more effectively exaggerate their body size through voice modulation than do females. Despite being detected as cheating more often than women, we show that men, who stand to gain relatively greater fitness benefits by successfully exaggerating their size^11,25,35^, shifted their formant frequencies more than did women. Men thus simulated a longer vocal tract and larger body size, and more effectively biased listeners to overestimate their height. From the receiver’s side, we also found that, when primed to the possibility of deception, male listeners were less susceptible than female listeners to deceptive signals produced by other men if they correctly detected them as exaggerating or attenuating their body size. The specificity of this effect to the second experiment suggests that male listeners are particularly attuned to the deceptive signals of other men when an explicit competitive context is induced. Together these findings support the prediction that pressure on listeners to counteract deception by recalibrating size judgements for deceptive signals may be maximised in the context of male–male competition. Finally, our finding that listeners can effectively gauge the relative heights of deceivers predicts an asymmetry in the impact of deception on male and female listeners^6^. Indeed, assuming all males exaggerate, females should retain the ability to rank relative male quality, which is crucial in mate choice. In contrast, males may overestimate the absolute size of exaggerating competitors when deception goes undetected. In male–male competition, the size and strength of a rival compared to oneself is critical^37^, and thus males may overvalue the cost of continued conflict. This sexual asymmetry is again consistent with the dominant view that male–male competition is the primary mechanism of selection on men’s sexually dimorphic traits^42^, including voice pitch^43^, and here, a probable key driver of size exaggeration.

Honesty has been identified in exaggerated vocal signals of other species, such as red deer^29,30,44^ and koalas^45,46^ who have both permanent and behavioural adaptations for size exaggeration and where physical constraints enforce reliability. Hypothetically this may lead receivers to adapt to deception by shifting their perceptual scale to exaggerated ranges. Here, we show that absolute deception remains effective, indicating that such perceptual shifts are only partial. We suggest that similar effectiveness may be predicted in nonhuman signalling systems where deception is behavioural and facultative, and especially where constraints enforce a degree of reliability in the signalling of interindividual differences.

Body size exaggeration is suspected in numerous species across a range of taxa;^6,8,19,26^ however, to our knowledge, its effectiveness in biasing listeners had never been established. While researchers often generalize animal models to humans, our work shows that studying the human animal can answer key questions about animal behaviour that are otherwise difficult to tackle (see also^47^). At the same time, humans are exceptionally complex. Beyond body size, human voice fundamental and formant frequencies predict a range of biologically and socially relevant traits, such as dominance, strength, and attractiveness^25,35,48^, that can interact cross-modally with visual and olfactory cues to influence listeners’ perceptions of the signaler. Voice modulation can also communicate motivation (e.g., aggressive intent) rather than physical traits per se^49,50^. Research into vocal deception of a wide range of traits^51^ and states^50^, particularly in multimodal real-world contexts^52^, is needed to further elucidate the functions and tangible consequences of deceit in complex social environments. Humans also possess an unpreceded capacity for volitional vocal control^21^ and an advanced theory of mind. While intentionality is not necessary for deceptive signalling to evolve (i.e., functional deception^5^), this has nevertheless led many researchers to suggest that ‘human communication is permeated with deceitʼ^6^, perhaps more so than the communication systems of other animals. Indeed, our results suggest that priming listeners to deception can substantially decrease its effectiveness and thus reduce potential costs of being deceived for cognisant listeners. Further comparative work is needed to elucidate the cognition of deception^8^, in humans and nonhuman animals.

## Methods

### Vocal stimuli

Vocal stimuli derived from 40 adult English speakers: 20 men (mean age 19.6 ± 2.4 sd) whose heights ranged from 161 cm to 187 cm (mean height 178.4 ± 7 cm sd) and 20 women (mean age 19.1 ± 1.6 sd) whose heights ranged from 147 to 185 cm (mean height 164.9 ± 7.9 sd), taken from a larger sample of vocal stimuli (see^38^). The stimuli were selected to be representative of a broad range of heights: the height distributions of male and female vocalisers closely parallel those observed in large cross-cultural samples of adults (men 178 ± 6.58 cm, *n* = 1334; women 165.96 ± 6.64 cm, *n* = 871)^34^. Vocalisers were recorded in an anechoic sound-controlled chamber with a Sennheiser MKH 800 cardioid condenser microphone at a distance of approximately 10 cm. Voice stimuli consisted of a series of monophthong vowels (/α/, /i/, /ɛ/, /o/, /u/). Each vocaliser produced the vowels in three conditions, beginning with a baseline ‘honest’ condition in which they spoke the vowels in their natural voice. They were then asked to reproduce the vowels while sounding physically large, and again while sounding physically small, in a counter-balanced order across participants^38^. No further instructions were given. Recordings were digitally encoded with an M-Audio Fast Track interface at a sampling rate of 96 kHz and 24-bit amplitude quantisation, and stored onto a computer as PCM WAV files. This procedure resulted in 120 vocal stimuli.

### Acoustic analysis

Fundamental frequency (*f*_o_) and the first four formant frequencies (*F*_1_–*F*_4_) were measured using the well-established autocorrelation algorithm (*f*_o_ range 30–500 Hz for men and 65–600 Hz for women) and Burg linear predictive coding algorithm (max formant 5000 Hz for men and 5500 Hz for women) in Praat acoustic software^40^. Formant measures were taken from the mean centre frequencies of each vowel and averaged within vocalisers and voice conditions. From *F*_1_ to *F*_4_ we computed formant spacing (∆*F*), a measure of the distance among adjacent formants, and apparent VTL^31,34^, an estimate of the length of the supralaryngeal vocal tract (Fig. 1), both of which explain the highest proportion of variance in height among humans^34^ and many other mammals^27^ within sex–age classes. Mean *f*_o_ measures were additionally transformed into equivalent rectangular bandwidth units (ERB, where Ei = 21.4*log_10_(0.00437*fi + 1)^53^ and formant measures were transformed into Bark units (where Zi = 26.81/(1 + 1960/fi) − 0.53)^54^. These quasi-logarithmic scales control for any difference between the physical and perceived properties of these frequencies. However, due to extremely strong collinearity between the two measures of mean *f*_o_ (Hz and ERB, *r* = 0.99) and among various formant measures (∆*F* and VTL in Hz and Bark, *r* = −0.99), we found virtually identical results regardless of which measure was used for each given vocal parameter, and thus, data are presented for ∆*F* and *f*_o_ in Hz only.

### Listeners

Two-hundred adult listeners took part in one of two psychoacoustic experiments. Sample sizes for Experiment 1 were determined prior to testing to achieve an average of 50 height judgements per vocal stimulus for a statistical power of 80%, in order to obtain a small-to-medium effect size in regressions between perceived and actual vocaliser height. While high inter-rated agreement (alphas > 0.80, *p*s < 0.001) among listeners is typically achieved with relatively small sample sizes (e.g., less than 15 listeners per sex for voice-based judgements of dominance or attractiveness^43^), earlier studies on human vocal communication of body size have generally failed to find significant correlations between perceived and actual height in one or both sexes of vocalisers with samples of fewer than 25 listeners per vocal stimulus^55–57^. In Experiment 2, tasks were conjoined, and thus participants rated all vocal stimuli (see Psychoacoustic playback experiments). English-speaking listeners were recruited via Amazon’s online recruitment platform, Mechanical Turk. The use of headphones was mandatory. In Experiment 1, three participants did not finish the study and were thus excluded from analyses. In the final sample (*n* = 97), 59 participants indicated male gender (aged 18–63, 9.2 sd), 36 indicated female gender (aged 18–63, 11.1 sd), and two indicated their gender as ‘other’. In Experiment 2, two participants provided random responses and were thus excluded from analyses. In the final sample (*n* = 98), 59 participants indicated male gender (aged 21–71, 9.7 sd) and 39 indicated female gender (aged 18–55, 9.4 sd). This research was approved by the Institutional Ethics Committee (C-REC; Certificates of approval: ER/KP292/11 and ER/REBY/12). All participants provided informed consent and were reimbursed monetarily at the recommended rate of $0.13 USD per minute^58^.

### Psychoacoustic playback experiments

Listeners completed a short demographic questionnaire and took part in one of the two psychoacoustic playback experiments, custom designed in Syntoolkit^59^, each involving two tasks: judging vocaliser height and detecting vocal size deception. In Experiment 1, listeners performed these two tasks in separate, consecutive blocks. In each task listeners rated the voices of a random sample of 10 male and 10 female vocalisers, in each of three voice conditions (honest, exaggerated, and attenuated size), resulting in 60 trials per task, or a total of 120 trials per listener. Height judgements preceded the deception detection task so as not to prime nor bias listeners toward contemplating deception when judging height, thus reflecting a more ecologically valid experimental design. Importantly, the same voice stimuli were presented in both tasks within listeners to allow for meaningful comparisons. In Experiment 2, an independent sample of listeners performed the same tasks; however, the tasks were now performed concurrently for each vocal stimulus. Thus, listeners first indicated whether or not they perceived a vocaliser as deceptively altering their voice to sound larger or smaller, and then judged the height of that same vocaliser, within the same experimental trial. Listeners judged all 20 male and 20 female vocalisers, in each of three voice conditions (honest, exaggerated, and attenuated size), for a total of 120 trials per listener. In both Experiments, listeners were presented with a single vocal stimulus on each trial. Voice stimuli were blocked by the sex of the vocaliser, and block order and stimulus presentation within each block were randomized. Listeners were instructed to wear headphones and not to adjust their volume settings throughout the experiment; this was verified during debriefing.

### Task 1: Judging height

To indicate the perceived height of a vocaliser, listeners used a vertical sliding bar, which appeared only after a voice stimulus finished playing. As the participant moved the cursor along the vertical sliding bar, the selected height was indicated in both metric (cm) and imperial (feet and inches) units. Maximum and minimum heights were labelled on the top and bottom of the scale, respectively, based on sex-specific distributions of heights in our samples, which correspond closely with those observed in the general population^34^. Thus, the centre of the sliding bar was set to 178 cm for men and 165 cm for women, with end points corresponding to three standard deviations above and below these means: a range of 156–198 cm for men and 144–186 cm for women.

### Task 2: Detecting deception

To indicate perceived vocal size deception or its absence, listeners were instructed to indicate whether they believed the vocaliser was speaking in their natural voice or changing their voice to sound physically smaller or larger than they actually are. These three options, presented as radial buttons, appeared only after the voice stimulus finished playing.

### Statistical analysis

A series of LMMs fit by restricted maximum likelihood estimation were used to examine listeners’ height judgements, correct detection of vocal size deception, and the influence of this detectable deception on height judgements. The key variable of interest (size deception: honest, attenuated, exaggerated) was entered in LMMs as a fixed variable. Vocaliser and listener IDs were always included as random variables with random intercepts in all models, and the sex of both vocaliser and listener were entered as fixed variables in omnibus models. Vocaliser sex consistently showed significant effects; therefore separate LMMs are reported for male and female vocalisers. Where there were no significant effects of listener sex (Experiment 1) listener data were pooled for all analyses; otherwise listener sex was retained in final models where applicable. Full model parameters of LMMs are detailed below each respective output table (see Supplementary Tables 2, 5, 7, 8, 9, 11, 12, 13 and 14). All means, confidence intervals (95% CI), and standard errors (SEM) reported in the paper derive from LMMs. Significant effects in LMMs were further examined using pairwise tests with Šidák correction for multiple comparisons. Multiple and simple linear regression models were employed to examine relationships among continuous variables. Where applicable, height judgements were averaged across listeners for each vocaliser and voice condition. Spearman’s rho (*r*_s_) correlation coefficients were used where data were nonnormally distributed or potentially nonlinear, and these statistics are reported in the paper, however Pearson’s *r* coefficients are given for comparison in Supplementary Tables 3, 4, and 6. Cook’s distances were calculated to identify influential outliers in simple regressions (where Di > 0.20), and the statistics reported in the paper exclude outliers. Removing or retaining outliers did not affect the direction and general pattern of relationships. We used an alpha level of 0.05 for all statistical tests, and all tests were two-tailed with the exception of simple regressions, where we had a priori directional predictions. Error bars in all figures represent standard errors of the mean (±SEM), whereas 95% CI are given in text. Statistical analyses were performed in SPSS 24 (IBM). Full statistical models and data are available in the supplementary files of this article (see Supplementary Data 1 and Supplementary Information).

### Ethics statement

This research was approved by the University of Sussex’s Life Sciences & Psychology Cluster-based Research Ethics Committee (C-REC; Certificates of approval: ER/KP292/11 and ER/REBY/12) and complies with the American Psychological Association’s Ethical Principles of Psychologists and Code of Conduct, including obtaining informed consent from all human participants.

### Reporting summary

Further information on research design is available in the Nature Research Reporting Summary linked to this article.

## Supplementary information

Supplementary Information

Peer Review File

Reporting Summary

Description of Additional Supplementary Files

Supplementary data 1

## Data Availability

All data generated or analysed during this study are included in this published article and its supplementary information files, including source data for all figures provided as a Source Data file. Datasets and voice stimuli (*n* = 120 audio WAV files) are also available on the Open Science Framework (https://osf.io/r7gzb/, 10.17605/OSF.IO/R7GZB). Source data are provided with this paper.

## Source data

Source Data

## Supplementary information

**Supplementary information** The online version contains supplementary material available at 10.1038/s41467-021-21008-7.
