# Supplementary material for: Efficacy in deceptive vocal exaggeration of human body size
Source: Nat Commun. 2021 Feb 12;12:968. doi: 10.1038/s41467-021-21008-7 (PMC7881139; doi:10.1038/s41467-021-21008-7)
Supplement: Supplementary file 1 — Supplementary Information [file 41467_2021_21008_MOESM1_ESM.pdf]

Supplementary Information for:

Efficacy in deceptive vocal exaggeration of human body size

Katarzyna Pisanski (kasiapisanski@gmail.com; ORCID 0000-0003-0992-2477)
David Reby (ORCID 0000-0001-9261-1711)

Supplementary Tables

Supplementary Table 1. Absolute acoustic parameters measured from honest and deceptive vocal signals, and percentage change during size deception

	Male vocalisers					Female vocalisers				
	Honest	Attenuating		Exaggerating		Honest	Attenuating		Exaggerating	
Voice Parameter	Mean	Mean	% change	Mean	%	Mean	Mean	%	Mean	%
Mean Δ*F*, Hz	1023.6 (2.4)	1054.0 (1.0)	3.0	954.9 (1.8)	-6.7	1195.3 (1.4)	1215.8 (1.7)	1.7	1161.0 (1.6)	-2.8
Mean Δ*F*, Bark	8.66 (0.01)	8.84 (0.01)	2.1	8.25 (0.01)	-4.7	9.62 (0.01)	9.73 (0.01)	1.1	9.50 (0.01)	-1.2
Mean VTL, cm	17.18 (0.04)	16.62 (0.012)	-3.3	18.40 (0.04)	7.1	14.66 (0.02)	14.42 (0.02)	-1.6	15.10 (0.02)	3.0
Mean f_o, Hz	117.3 (0.7)	121.0 (0.6)	3.2	109.4 (0.5)	-6.7	213.8 (0.7)	231.3 (1.0)	8.2	203.21 (0.9)	-5.0
Mean f_o, ERB	3.83 (0.02)	3.93 (0.01)	2.6	3.62 (0.01)	-5.5	6.12 (0.01)	6.47 (0.02)	5.7	5.89 (0.02)	-3.8

Mean absolute values (standard error of the mean, ±SEM), and percentage change (%) from baseline (honest condition) during vocal size attenuation and exaggeration, where negative percentages indicate a decrease from baseline. Note: Δ*F* = formant spacing; VTL = apparent vocal tract length; f_o = fundamental frequency in Hertz (Hz). Psychoacoustic auditory rescaling to equivalent rectangular bandwidths (ERBs, where $E_i = 21.4*\log_{10}(0.00437*f_i+1)]$[1]) and Bark units, where $Z_i = 26.81/(1+1960/f_i) - 0.53$ [2]. Measures based on *n*=40 vocalisers (20 males, 20 females), 120 vocal stimuli (20 per sex, per size condition).

Supplementary Table 2. Linear Mixed Models: Sex differences in voice frequency shifts

Source	*df1, df2*	*F*	*P*	*df1, df2*	*F*	*P*
**Final Models (a)**		Δ*F*			f_o	
Intercept	1, 33	24.5	<.001	1, 37	12.8	.001
Vocaliser sex (exaggerating)	1, 33	5.5	.025	1, 37	0.2	.630
Intercept	1, 32	4.7	.037	1, 37	11.5	.002
Vocaliser sex (attenuating)	1, 32	0.1	.842	1, 37	3.6	.064
**Final Models (b)**		Δ*F*				
Vocaliser sex (exaggerating)	1, 30	3.6	.067			
**Omnibus Models**		Δ*F*			f_o	
Intercept	1, 36.4	3.6	.064	1, 37	0.1	.816
Size deception	2, 32.9	57.1	<.001	1, 37	38.0	<.001
Vocaliser sex	1, 36.3	2.0	.168	1, 37	1.0	.335
Size deception * vocaliser sex	1, 32.9	6.1	.019	1, 37	5.0	.032

Linear mixed models (LMMs). All models, dependent variable: magnitude of voice frequency shift (difference from 'honest' baseline) for each voice frequency parameter (note that analogous results are obtained on ERB and Bark psychoacoustic scales); random variable (with intercept): vocaliser ID. Final models (a), fixed variable: sex of vocaliser (conducted separately for each deceptive size condition); Omnibus model, fixed variables: size deception * vocaliser sex; Final models (b), same as 'a' controlling for vocaliser actual height as a fixed covariate. Significant effects in LMMs were further examined using pairwise tests with Šidák correction for multiple comparisons. All tests two-tailed, alpha 0.05. *n*=40 vocalisers (20 males, 20 females), 120 vocal stimuli (20 per sex, per size condition).

Supplementary Table 3. Relationships between voice frequency shifts and vocaliser height

Correlation Coefficient	Shift in Voice Parameter	Male vocalisers Size Deception: Attenuating *r* (*n, p*)	Male vocalisers Size Deception: Exaggerating *r* (*n, p*)	Female vocalisers Size Deception: Attenuating *r* (*n, p*)	Female vocalisers Size Deception: Exaggerating *r* (*n, p*)
r_s	Mean Δ*F* (Hz)	0.34† (15, .108)	-0.55* (15, .017)	0.10 (16, .360)	0.29 (14, .154)
	Mean Δ*F*, Bark	0.34† (15, .108)	-0.55* (15, .017)	0.10 (16, .360)	0.29 (14, .154)
	Mean f_o (Hz)	0.19 (17, .233)	0.05 (16, .427)	-0.09 (19, .352)	-0.29 (17, .126)
	Mean f_o, ERB	0.19 (17, .233)	0.03 (18, .459)	-0.04 (18, .348)	-0.29 (19, .119)
r	Mean Δ*F* (Hz)	0.37† (16, .077)	-0.56* (16, .013)	0.15 (17, .285)	0.26 (16, .167)
	Mean Δ*F*, Bark	0.37 (16, .078)	-0.56* (16, .013)	0.15 (17, .284)	0.26 (16, .165)
	Mean f_o (Hz)	0.28 (18, .130)	-0.14 (18, .292)	-0.13 (19, .292)	-0.28 (18, .130)
	Mean f_o, ERB	0.28 (18, .129)	0.01 (17, .448)	-0.14 (19, .279)	-0.27 (18, .139)

Spearman *rho* (r_s) and Pearson's *r* correlations. Note: Δ*F* = formant spacing; f_o = fundamental frequency. Psychoacoustic auditory rescaling to equivalent rectangular bandwidths (ERBs, where $E_i = 21.4*\log_{10}(0.00437*f_i+1)$] [1]) and Bark units, where $Zi = 26.81/(1+1960/f_i) - 0.53$ [2]. Significant effects indicated with *p<.05, †p<.010, one-tailed, *n*=40 vocalisers, 15-19 per correlation where Cook's *Di* < 0.20. Exact *n* and *p* values are given, respectively, in brackets to the right of each correlation coefficient.

Supplementary Table 4. Relationships between absolute voice frequencies and vocaliser height

Correlation Coefficient	Voice Parameter	Male vocalisers: Honest *r* (*n*, *p*)	Male vocalisers: Size Deception: Attenuating *r* (*n*, *p*)	Male vocalisers: Size Deception: Exaggerating *r* (*n*, *p*)	Female vocalisers: Honest *r* (*n*, *p*)	Female vocalisers: Size Deception: Attenuating *r* (*n*, *p*)	Female vocalisers: Size Deception: Exaggerating *r* (*n*, *p*)
r_s	Mean Δ*F* (Hz)	-0.37† (18, .070)	-0.21 (17, .220)	-0.76** (18, <.001)	-0.63** (19, .002)	-0.41* (16, .042)	-0.31 (18, .110)
	Mean f_o (Hz)	.17 (19, .245)	0.35 (20, .065)	0.05 (18, .427)	0.17 (18, .254)	0.14 (18, .285)	0.19 (17, .234)
r	Mean Δ*F* (Hz)	-0.35† (18, .080)	-0.25 (17, .170)	-0.75** (18, <.001)	-0.69** (19, .001)	-0.44* (16, .045)	-0.13 (15, .320)
	Mean f_o (Hz)	0.23 (19, .172)	0.39† (20, .053)	0.10 (18, .354)	0.24 (18, .166)	0.22 (18, .196)	0.26 (17, .154)

Spearman *rho* (r_s) and Pearson's *r* correlations. Note: Δ*F* = formant spacing; f_o = fundamental frequency. Significant effects indicated with ***p*<.001, **p*<.05, †*p*<.010, one-tailed, *n*=40 vocalisers, 16-20 per correlation where Cook's *Di* < 0.20. Exact *n* and *p* values are given, respectively, in brackets below each correlation coefficient.

Supplementary Table 5. Linear Mixed Models: Listeners' height judgments (Experiment 1)

Source	*df1, df2*	*F*	*P*	*df1, df2*	*F*	*P*
**Final Models**	Male vocalisers			Female vocalisers		
Intercept	1, 969	5.1	.024	1, 969	10.2	.001
Size deception	2, 1938.0	299.2	<.001	2, 1938.0	174.3	<.001
**Omnibus Model**	Both vocaliser sexes					
Intercept	1, 1936.5	13.8	<.001			
Size deception	2, 3872.0	439.2	<.001			
Vocaliser sex	1, 1936.5	0.7	.392			
Size deception * vocaliser sex	2, 3872.0	9.9	<.001			
Listener sex	1, 1945.4	0.1	.866			
Size deception * listener sex	2, 3872.4	0.2	.839			
Vocaliser sex * listener sex	1, 1945.4	0.4	.519			
Size deception * vocaliser sex * listener sex	2, 3872.4	0.1	.911			

Linear mixed models (LMMs). All models, dependent variable: Error in height judgments (difference perceived – actual vocaliser height); random variables (with intercept): listener ID * vocaliser ID. Final models, fixed variable: size deception (split by vocaliser sex). Omnibus model, fixed variables: size deception * vocaliser sex * listener sex. Significant effects in LMMs were further examined using pairwise tests with Šidák correction for multiple comparisons. All tests two-tailed, alpha 0.05. All data derive from Experiment 1, based on 120 vocal stimuli produced by *n*=40 vocalisers (20 males, 20 females) in each of three size conditions (honest, attenuating, exaggerating) and judged by *n*=97 listeners, where each vocal stimulus was rated by an average of 50 listeners (see Methods).

Supplementary Table 6. Relationships between voice frequencies and perceived vocaliser height (Experiment 1)

		Male vocalisers			Female vocalisers		
			Size Deception			Size Deception	
Correlation Coefficient	Voice Parameter	Honest *r* (*n, p*)	Attenuating *r* (*n, p*)	Exaggerating *r* (*n, p*)	Honest *r* (*n, p*)	Attenuating *r* (*n, p*)	Exaggerating *r* (*n, p*)
r_s	Mean ΔF (Hz)	-0.29† (17, .128)	0.16 (17, .264)	-0.59** (20, .003)	-0.66** (19, .001)	-0.35† (14, .113)	-0.74** (15, .001)
	Mean f_o (Hz)	-0.45* (18, .030)	-0.35† (19, .074)	-0.74** (20, <.001)	-0.46* (19, .024)	-0.40* (17, .045)	-0.49* (18, .020)
r	Mean ΔF (Hz)	-.23 (17, .184)	0.22 (17, .200)	-0.53** (20, .009)	-0.66** (19, .001)	-0.46† (14, .050)	-0.78** (15, <.001)
	Mean f_o (Hz)	-.30 (18, .113)	-0.53* (17, .014)	-0.76** (19, <.001)	-0.38† (18, .058)	-0.59** (20, .003)	-0.61** (18, .003)

Spearman *rho* (r_s) and Pearson's *r* correlations. Note: ΔF = formant spacing; f_o = fundamental frequency. Significant effects indicated with ***p*<.001, **p*<.05, †*p*<.010, one-tailed, *n*=40 vocalisers, 14-19 per correlation where Cook's *Di* < 0.20. Exact *n* and *p* values are given, respectively, in brackets below each correlation coefficient.

Supplementary Table 7. Linear Mixed Models: Listeners' detection of size deception (Experiment 1)

Source	*df1, df2*	*F*	*P*	*df1, df2*	*F*	*P*
**Final Models (a)**	Male vocalisers			Female vocalisers		
Intercept	1, 2907	3381.5	<.001	1, 2907	2670.2	<.001
Size deception	2, 2907	15.7	<.001	2, 2907	28.7	<.001
**Final Models (b)**	Both vocaliser sexes					
Intercept	1, 1938	2754.2	<.001			
Vocaliser sex (honest)	2, 1938	3.1	.08			
Intercept	1, 1938	1824.3	<.001			
Vocaliser sex (exaggerating)	2, 1938	6.7	<.01			
Intercept	1, 1938	1551.5	<.001			
Vocaliser sex (attenuating)	2, 1938	14.5	<.001			
**Omnibus Model**	Both vocaliser sexes					
Intercept	1, 5808.0	5756.0	<.001			
Size deception	2, 5808.0	41.0	<.001			
Vocaliser sex	1, 5808.0	23.8	<.001			
Size deception * vocaliser sex	2, 5808.0	1.0	.362			
Listener sex	1, 5808.0	3.2	.075			
Size deception * listener sex	2, 5808.0	1.3	.262			
Vocaliser sex * listener sex	1, 5808.0	1.6	.200			
Size deception * vocaliser sex * listener sex	2, 5808.0	0.4	.693			

Linear mixed models (LMMs). All models, dependent variable: Correct detection of size deception (*correctly identified* as honest, exaggerating, or attenuating size); random variables (with intercept): listener ID * vocaliser ID. Final models a, fixed variable: size deception (split by vocaliser sex); Final models b, fixed variable: vocaliser sex (split by size deception condition); Omnibus model, fixed variables: size deception * vocaliser sex * listener sex. Significant effects in LMMs were further examined using pairwise tests with Šidák correction for multiple comparisons. All tests two-tailed, alpha 0.05. All data derive from Experiment 1, based on 120 vocal stimuli produced by *n*=40 vocalisers (20 males, 20 females) in each of three size conditions (honest, attenuating, exaggerating) and judged by *n*=97 listeners, where each vocal stimulus was rated by an average of 50 listeners (see Methods).

Supplementary Table 8. Linear Mixed Models: Effect of *unprimed* deception detection on 'error' in height judgments (difference between perceived and actual vocaliser height) (Experiment 1)

Source	*df1, df2*	*F*	*P*	*df1, df2*	*F*	*P*
**Final Models**	Male vocalisers			Female vocalisers		
Intercept	1, 974.8	6.2	.013	1, 978.5	11.1	.001
Size deception	2, 1940.9	303.7	<.001	2, 1944.3	188.5	<.001
Deception detection	1, 2264.6	0.9	.334	1, 2170.2	1.2	.279
Size deception * deception detection	2, 2325	15.0	<.001	2, 2344.8	16.6	<.001
**Omnibus Model**	Both vocaliser sexes					
Intercept	1, 1905.5	13.3	<.001			
Size deception	2, 3764.6	436.1	<.001			
Deception detection	1, 4300.8	1.8	.179			
Vocaliser sex	1, 1905.4	0.8	.369			
Listener Sex	1, 1914.5	0.2	.757			
Size deception * deception detection	4, 4528.9	31.1	<.001			
Size deception * vocaliser sex	2, 3764.6	8.1	<.001			
Size deception * listener sex	2, 3765.0	0.2	.994			
Deception detection * vocaliser sex	1, 4300.8	0.4	.503			
Deception detection * listener sex	1, 4300.9	0.2	.629			
Speaker sex * listener sex	1, 1914.5	0.2	.664			
Size deception * deception detection * vocaliser sex	2, 4528.9	1.4	.258			
Size deception * deception detection * listener sex	2, 4528.8	0.2	.826			
Size deception * vocaliser sex * listener sex	2, 3765.0	0.2	.832			
Deception detection * vocaliser sex * listener sex	1, 4300.9	3.2	.074			
Size deception * deception detection * vocaliser sex * listener sex	2, 4528.8	0.8	.436			

Linear mixed models (LMMs). All models, dependent variable: Error in height judgments (difference between perceived and actual vocaliser height); random variables (with intercept): listener ID * vocaliser ID. Final models, fixed variables: size deception * deception detection (split by vocaliser sex). Omnibus model, fixed variables: size deception * deception detection * vocaliser sex * listener sex. Significant effects in LMMs were further examined using pairwise tests with Šidák correction for multiple comparisons. All tests two-tailed, alpha 0.05. All data derive from Experiment 1, based on 120 vocal stimuli produced by *n*=40 vocalisers (20 males, 20 females) in each of three size conditions (honest, attenuating, exaggerating) and judged by *n*=97 listeners, where each vocal stimulus was rated by an average of 50 listeners (see Methods).

Supplementary Table 9. Linear Mixed Models: Effect of *unprimed* deception detection on 'deception gain' (difference between perceived height from deceptive vocal signals and perceived height from honest vocal signals) (Experiment 1)

Source	*df1, df2*	*F*	*P*	*df1, df2*	*F*	*P*
**Final Models**	Male vocalisers			Female vocalisers		
Intercept	1, 967.9	5.8	.017	1, 986.1	2.0	.159
Size deception	1, 967.1	502.8	<.001	1, 989.2	348.2	<.001
Deception detection	1, 1678.6	0.1	.890	1, 1482.9	0.1	.777
Size deception * deception detection	1, 1785.1	18.3	<.001	1, 1758.9	26.4	<.001
**Omnibus Model**	Both vocaliser sexes					
Intercept	1, 1904.4	0.2	.644			
Size deception	1, 1900.2	768.1	<.001			
Deception detection	1, 3049.2	0.1	.817			
Vocaliser sex	1, 1904.4	6.8	.009			
Listener Sex	1, 1904.4	0.1	.861			
Size deception * deception detection	1, 3453.3	45.6	<.001			
Size deception * vocaliser sex	1, 1900.2	8.1	.005			
Size deception * listener sex	1, 1900.2	0.1	.948			
Deception detection * vocaliser sex	1,3049.2	0.4	.551			
Deception detection * listener sex	1, 3049.2	0.1	.908			
Speaker sex * listener sex	1, 1904.4	0.1	.840			
Size deception * deception detection * vocaliser sex	1, 3453.3	0.3	.606			
Size deception * deception detection * listener sex	1, 3453.3	0.3	.602			
Size deception * vocaliser sex * listener sex	1, 1900.2	0.3	.578			
Deception detection * vocaliser sex * listener sex	1, 3049.2	4.1	.054			
Size deception * deception detection * vocaliser sex * listener sex	1, 3453.3	0.1	.883			

Linear mixed models (LMMs). All models, dependent variable: 'Detection gain' in height judgments (difference between perceived height from deceptive signals and perceived height from honest signals); random variables (with intercept): listener ID * vocaliser ID. Final models, fixed variables: size deception * deception detection (split by vocaliser sex). Omnibus model, fixed variables: size deception * deception detection * vocaliser sex * listener sex. Significant effects in LMMs were further examined using pairwise tests with Šidák correction for multiple comparisons. All tests two-tailed, alpha 0.05. All data derive from Experiment 1, based on 120 vocal stimuli produced by *n*=40 vocalisers (20 males, 20 females) in each of three size conditions (honest, attenuating, exaggerating) and judged by *n*=97 listeners, where each vocal stimulus was rated by an average of 50 listeners (see Methods).

Supplementary Table 10. Magnitude of voice frequency shifts as a function of correct detection of deception (Experiment 1)

Shifted Voice Parameter	Detection of Deception	Male vocalisers		Female vocalisers	
		Attenuating	Exaggerating	Attenuating	Exaggerating
Mean Δ*F*, Hz	Incorrect	36.8 (2.5)	-66.4 (2.7)	21.3 (1.4)	-14.0 (2.3)
	Correct	13.2 (2.5)	-70.8 (3.2)	15.7 (2.0)	-44.9 (2.2)
Mean f_o, Hz	Incorrect	6.4 (0.5)	-3.1 (0.5)	16.5 (0.8)	-8.6 (0.6)
	Correct	7.5 (0.5)	-3.4 (0.5)	19.0 (1.2)	-13.0 (0.9)

Means (standard error of the mean, ±SEM), indicating the magnitude of voice frequency shifts (difference from 'honest' baseline), where positive values indicate raised frequencies, and negative values indicate lowered frequencies. Note: Δ*F* = formant spacing; f_o = fundamental frequency in Hertz (Hz). Based on *n*=40 vocalisers (20 males, 20 females), 120 vocal stimuli (20 per sex, per size condition).

Supplementary Table 11. Linear Mixed Models: Listeners' height judgments (Experiment 2)

Source	*df1, df2*	*F*	*P*	*df1, df2*	*F*	*P*
**Final Models**	Male vocalisers			Female vocalisers		
Intercept	1, 1987	0.1	.955	1, 1978	10.5	.001
Size deception	2, 3956	126.2	<.001	2, 3956	84.9	<.001
Listener sex	1, 1978	13.7	<.001	1, 1978	2.0	.156
Size deception * listener sex	2, 3956	6.2	.002	2, 3956	0.1	.996
**Omnibus Model**	Both vocaliser sexes					
Intercept	1, 3956	5.4	.020			
Size deception	2, 7912	205.0	<.001			
Vocaliser sex	1, 3956	5.8	.016			
Size deception * vocaliser sex	2, 7912	7.3	.001			
Listener sex	1, 3956	12.7	<.001			
Size deception * listener sex	2, 7912	3.1	.045			
Vocaliser sex * listener sex	1, 3956	2.3	.133			
Size deception * vocaliser sex * listener sex	2, 7912	3.3	.039			

Linear mixed models (LMMs). All models, dependent variable: Error in height judgments (difference perceived – actual vocaliser height); random variables (with intercept): listener ID * vocaliser ID. Final models, fixed variables: size deception * listener sex (split by vocaliser sex). Omnibus model, fixed variables: size deception * vocaliser sex * listener sex. Significant effects in LMMs were further examined using pairwise tests with Šidák correction for multiple comparisons. All tests two-tailed, alpha 0.05. All data derive from Experiment 2 based on 120 vocal stimuli produced by *n*=40 vocalisers (20 males, 20 females) in each of three size conditions (honest, attenuating, exaggerating) and judged by *n*=98 listeners, each of whom rated all vocal stimuli (see Methods). Analogous to Experiment 1 LMMs presented in Supplementary Table 5 above.

Supplementary Table 12. Linear Mixed Models: Listeners' detection of size deception (Experiment 2)

Source	*df1, df2*	*F*	*P*	*df1, df2*	*F*	*P*
**Final Models (a)**		Male vocalisers			Female vocalisers	
Intercept	1, 5934	6457.8	<.001	1, 5934	5271.5	<.001
Size deception	2, 5934	120.4	<.001	2, 5934	158.0	<.001
Listener sex	1, 5934	1.3	.248	1, 5934	0.9	.341
Size deception * listener sex	2, 5934	8.3	<.001	2, 5934	5.7	.003
**Final Models (b)**		Both vocaliser sexes				
Intercept	1, 3958	7373.7	<.001			
Vocaliser sex (honest)	1, 3958	8.0	.005			
Intercept	1, 3958	3034.3	<.001			
Vocaliser sex (exaggerating)	1, 3958	5.8	.016			
Intercept	1, 3958	2632.2	<.001			
Vocaliser sex (attenuating)	1, 3958	32.2	<.001			
**Omnibus Model**		Both vocaliser sexes				
Intercept	1, 11868	11703.1	<.001			
Size deception	2, 11868	275.5	<.001			
Vocaliser sex	1, 11868	34.2	<.001			
Size deception * vocaliser sex	2, 11868	2.7	.065			
Listener sex	1, 11868	0.1	.882			
Size deception * listener sex	2, 11868	12.2	<.001			
Vocaliser sex * listener sex	1, 11868	2.2	.136			
Size deception * vocaliser sex * listener sex	2, 11868	1.8	.172			

Linear mixed models (LMMs). All models, dependent variable: Correct detection of size deception (*correctly identified* as honest, exaggerating, or attenuating size); random variables (with intercept): listener ID * vocaliser ID. Final models a, fixed variables: size deception * listener sex (split by vocaliser sex); Final models b, fixed variable: vocaliser sex (split by size deception condition). Omnibus model, fixed variables: size deception * vocaliser sex * listener sex. Significant effects in LMMs were further examined using pairwise tests with Šidák correction for multiple comparisons. All tests two-tailed, alpha 0.05. All data derive from Experiment 2 based on 120 vocal stimuli produced by *n*=40 vocalisers (20 males, 20 females) in each of three size conditions (honest, attenuating, exaggerating) and judged by *n*=98 listeners, each of whom rated all vocal stimuli (see Methods). Analogous to Experiment 1 LMMs presented in Supplementary Table 7 above.

Supplementary Table 13. Linear Mixed Models: Effect of *primed* deception detection on 'error' in height judgments (difference between perceived and actual vocaliser height) (Experiment 2)

Source	*df1, df2*	*F*	*P*	*df1, df2*	*F*	*P*
**Final Models (a)**	Male vocalisers			Female vocalisers		
Intercept	1, 2018.0	1.8	.181	1, 2026.7	13.7	<.001
Size deception	2, 3986.4	112.1	<.001	2, 3992.1	63.8	<.001
Deception detection	1, 4470.5	1.7	.194	1, 4314.6	0.2	.657
Size deception * deception detection	2, 4634.1	35.3	<.001	2, 4573.1	18.4	<.001
**Final Models (b)**	Male vocalisers			Female vocalisers		
Intercept (honest)	1, 1976.0	8.0	.005	1, 1976.0	8.5	.004
Deception detection	1, 1976.0	22.4	<.001	1, 1976.0	13.2	.<.001
Listener sex	1, 1976.0	10.9	<.001	1, 1976.0	0.7	.387
Deception detection * listener sex	1, 1976.0	0.1	.905	1, 1976.0	2.8	.093
Intercept (exaggerating)	1, 1976.0	45.3	<.001	1, 1976.0	2.0	.156
Deception detection	1, 1976.0	85.9	<.001	1, 1976.0	8.3	.004
Listener sex	1, 1976.0	25.2	<.001	1, 1976.0	1.2	.265
Deception detection * listener sex	1, 1976.0	0.1	.763	1, 1976.0	0.2	.629
Intercept (attenuating)	1, 1976.0	36.0	<.001	1, 1976.0	50.3	<.001
Deception detection	1, 1976.0	3.2	.076	1, 1976.0	15.2	<.001
Listener sex	1, 1976.0	2.3	.127	1, 1976.0	0.4	.546
Deception detection * listener sex	1, 1976.0	7.3	.007	1, 1976.0	1.9	.172
**Omnibus Model**	Both vocaliser sexes					
Intercept	1, 4032.2	7.7	.006			
Size deception	2, 7962.7	171.4	<.001			
Deception detection	1, 8782.1	0.2	.641			
Vocaliser sex	1, 4032.2	4.5	.033			
Listener Sex	1, 4032.2	11.9	.001			
Size deception * deception detection	2, 9187.3	47.3	<.001			
Size deception * vocaliser sex	2, 7962.7	9.27	<.001			
Size deception * listener sex	2, 7962.7	3.9	.020			
Deception detection * vocaliser sex	1, 8782.1	2.0	.155			
Deception detection * listener sex	1, 8782.1	0.1	.931			
Speaker sex * listener sex	1, 4032.2	2.5	.114			
Size deception * deception detection * vocaliser sex	2, 9187.3	3.5	.029			
Size deception * deception detection * listener sex	2, 9187.3	3.8	.023			
Size deception * vocaliser sex * listener sex	2, 7962.7	4.1	.017			
Deception detection * vocaliser sex * listener sex	1, 8782.1	1.2	.266			
Size deception * deception detection * vocaliser sex * listener sex	2, 9187.3	4.3	.014			

Linear mixed models (LMMs). All models, dependent variable: Error in height judgments (difference between perceived and actual vocaliser height); random variables (with intercept): listener ID * vocaliser ID. Final models a, fixed variables: size deception * deception detection (split by vocaliser sex). Final models b, fixed variables: deception detection * listener sex (split by vocaliser sex and size deception condition). Omnibus model, fixed variables: size deception * deception detection * vocaliser sex * listener sex. Significant effects examined with Šidák corrected pairwise comparisons. All tests two-tailed, alpha 0.05. All data derive from Experiment 2 based on 120 vocal stimuli produced by *n*=40 vocalisers (20 males, 20 females) in each of three size conditions (honest, attenuating, exaggerating) and judged by *n*=98 listeners, each of whom rated all vocal stimuli (see Methods). Analogous to Experiment 1 LMMs in Supplementary Table 8 above.

Supplementary Table 14. Linear Mixed Models: Effect of *primed* deception detection on 'deception gain' (difference between perceived height from deceptive vocal signals and perceived height from honest vocal signals) (Experiment 2)

Source	*df1, df2*	*F*	*P*	*df1, df2*	*F*	*P*
**Final Models (a)**	Male vocalisers			Female vocalisers		
Intercept	1, 1993.8	7.6	.006	1, 2077.7	3.1	.081
Size deception	1, 1992.8	186.1	<.001	1, 2082.5	119.7	<.001
Deception detection	1, 3426.0	5.2	.023	1, 3189.5	1.8	.178
Size deception * deception detection	1, 3422.1	27.1	<.001	1, 3469.6	24.2	<.001
**Final Models (b)**	Male vocalisers			Female vocalisers		
Intercept (exaggerating)	1, 1976.0	103.2	<.001	1, 1976.0	19.4	<.001
Deception detection	1, 1976.0	18.4	<.001	1, 1976.0	17.2	<.001
Listener sex	1, 1976.0	6.2	.013	1, 1976.0	.009	.924
Deception detection * listener sex	1, 1976.0	5.0	.025	1, 1976.0	.262	.609
Intercept (attenuating)	1, 1976.0	27.7	<.001	1, 1976.0	52.2	<.001
Deception detection	1, 1976.0	6.4	.012	1, 1976.0	5.1	.025
Listener sex	1, 1976.0	3.1	.077	1, 1976.0	0.2	.687
Deception detection * listener sex	1, 1976.0	8.3	.004	1, 1976.0	2.9	.087
**Omnibus Model**	Both vocaliser sexes					
Intercept	1, 4056.6	0.6	.456			
Size deception	1, 4060.2	309.3	<.001			
Deception detection	1, 6568.7	6.9	.009			
Vocaliser sex	1, 4056.6	10.4	.001			
Listener Sex	1, 4056.6	0.1	.714			
Size deception * deception detection	1, 6923.4	43.9	<.001			
Size deception * vocaliser sex	1, 4060.2	6.3	.012			
Size deception * listener sex	1, 4060.2	6.3	.012			
Deception detection * vocaliser sex	1, 6568.7	0.9	.354			
Deception detection * listener sex	1, 6568.7	0.1	.744			
Speaker sex * listener sex	1, 4056.6	0.1	.846			
Size deception * deception detection * vocaliser sex	1, 6923.4	0.2	.646			
Size deception * deception detection * listener sex	1, 6923.4	2.8	.092			
Size deception * vocaliser sex * listener sex	1, 4060.2	8.2	.004			
Deception detection * vocaliser sex * listener sex	1, 6568.7	1.8	.175			
Size deception * deception detection * vocaliser sex * listener sex	1, 6923.4	10.4	.001			

Linear mixed models (LMMs). All models, dependent variable: 'Detection gain' in height judgments (difference between perceived height from deceptive signals and perceived height from honest signals); random variables (with intercept): listener ID * vocaliser ID. Final models a, fixed variables: size deception * deception detection (split by vocaliser sex). Final models b, fixed variables: deception detection * listener sex (split by vocaliser sex and size deception condition). Omnibus model, fixed variables: size deception * deception detection * vocaliser sex * listener sex. Significant effects examined with Šidák corrected pairwise comparisons. All tests two-tailed, alpha 0.05. All data derive from Experiment 2 based on 120 vocal stimuli produced by *n*=40 vocalisers (20 males, 20 females) in each of three size conditions (honest, attenuating, exaggerating) and judged by *n*=98 listeners, each of whom rated all vocal stimuli (see Methods). Analogous to Experiment 1 LMMs presented in Supplementary Table 9 above.

Supplementary Figures

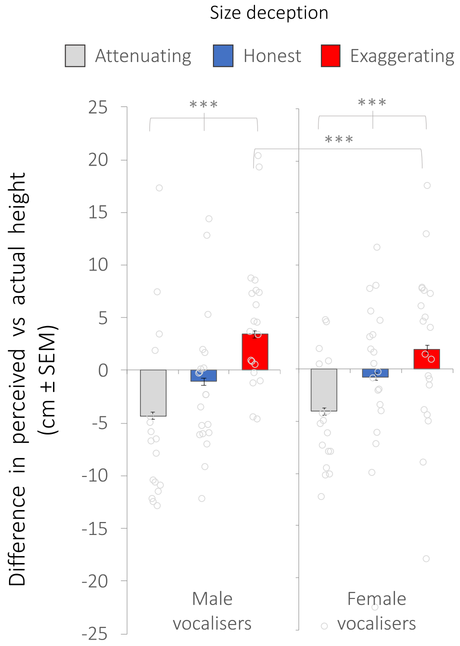

Supplementary Figure 1. Vocal size deception biases judgments of body size (Experiment 1) – bar graphs with overlaid dot plots. Bias in height judgments shown as the mean difference (±SEM) between perceived and actual heights of vocalisers, in cm, for honest vocal signals (central blue bars) and deceptive vocal signals (attenuating = grey bars, exaggerating = red bars), where 0 indicates accurate height judgments, positive values indicate overestimation and negative values indicate underestimation. Estimated marginal means and pairwise comparisons derive from linear mixed models, LMMs (see Supplementary Table 5), where all *** $p<.001$ following Šidák correction for multiple comparisons. Tests are two-tailed. Error bars represent standard errors of the mean, ±SEM. All data derive from Experiment 1, based on 120 vocal stimuli produced by $n=40$ vocalisers (20 males, 20 females) in each of three size conditions (honest, attenuating, exaggerating) and judged by $n=97$ listeners, where each vocal stimulus was rated by an average of 50 listeners (see Methods). Overlaid dot plots show the full distribution across vocalisers, each dot representing a single vocaliser with raw difference scores averaged across listeners and within vocalisers of each sex. Source data are provided as a Source Data file.

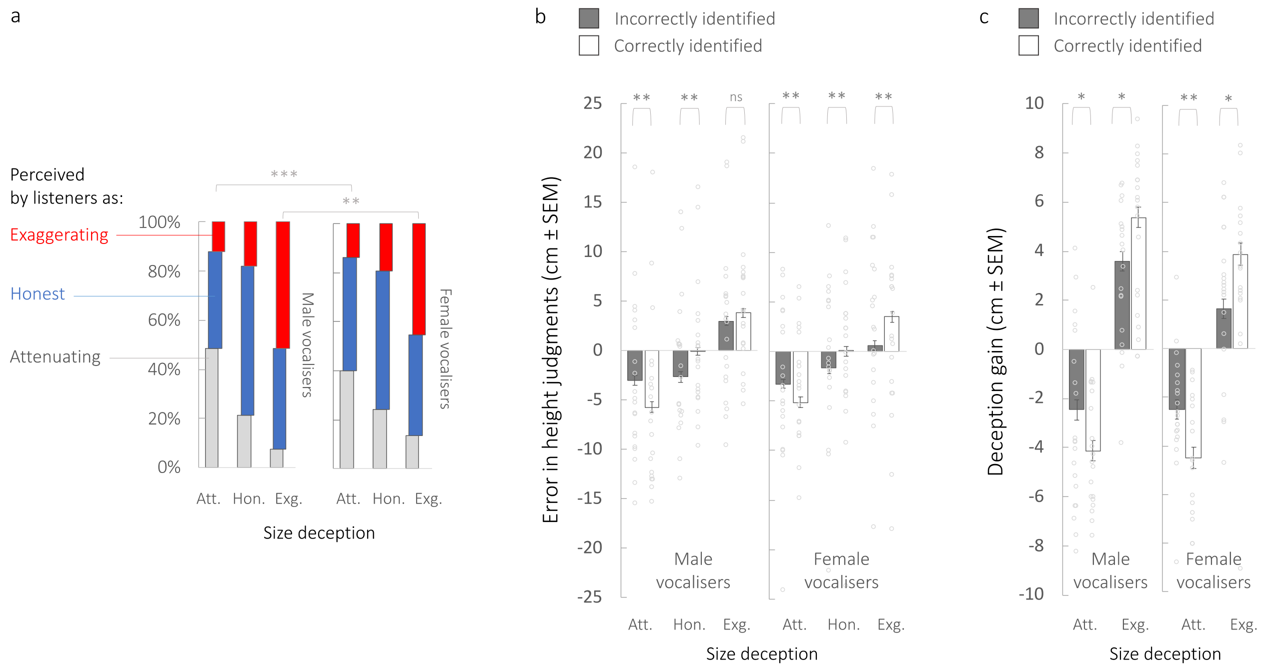

Supplementary Figure 2. Listeners can detect deception, but remain deceived by deceptive signals (Experiment 1) – bar graphs with overlaid dot plots. (a) Percentages of vocalisers that listeners perceived as deceptively exaggerating (red bars) or attenuating (light grey bars) their size, or as producing honest vocal signals (blue bars, center) are shown along the y-axis as a function of intended size deception indicated along the x-axis. Estimated marginal means and pairwise comparisons derive from LMMs (see Supplementary Table 7), where *** p<.001, ** p<.01, following Šidák correction for multiple comparisons. Tests are two-tailed. (b-c) Bias in listeners' size assessments as a function of whether a listener failed to detect (dark grey bars) or correctly detected (white bars) a vocal signal as deceptive or honest, where panel b shows 'error' in height judgments (mean difference between perceived vs actual heights of vocalisers), and panel c shows 'deception gain' in height judgments (mean difference between perceived height from honest signals and perceived height from deceptive signals). Estimated marginal means and pairwise comparisons derive from LMMs (see Supplementary Tables 8 and 9), ** p<.01, * p<.05 following Šidák correction. Tests are two-tailed. Error bars, ±SEM. Overlaid dot plots show the full distribution across vocalisers, each dot representing a single vocaliser (n=40, 20 males, 20 females) with raw difference scores averaged across listeners (on average 50 per datapoint) and within vocalisers of each sex. Acronyms: Att. (attenuating); Hon. (honest); Exg. (exaggerating). All data derive from Experiment 1, based on 120 vocal stimuli produced by n=40 vocalisers (20 males, 20 females) in each of three size conditions (honest, attenuating, exaggerating) and judged by n=97 listeners, where each vocal stimulus was rated by an average of 50 listeners (see Methods). Source data are provided as a Source Data file.

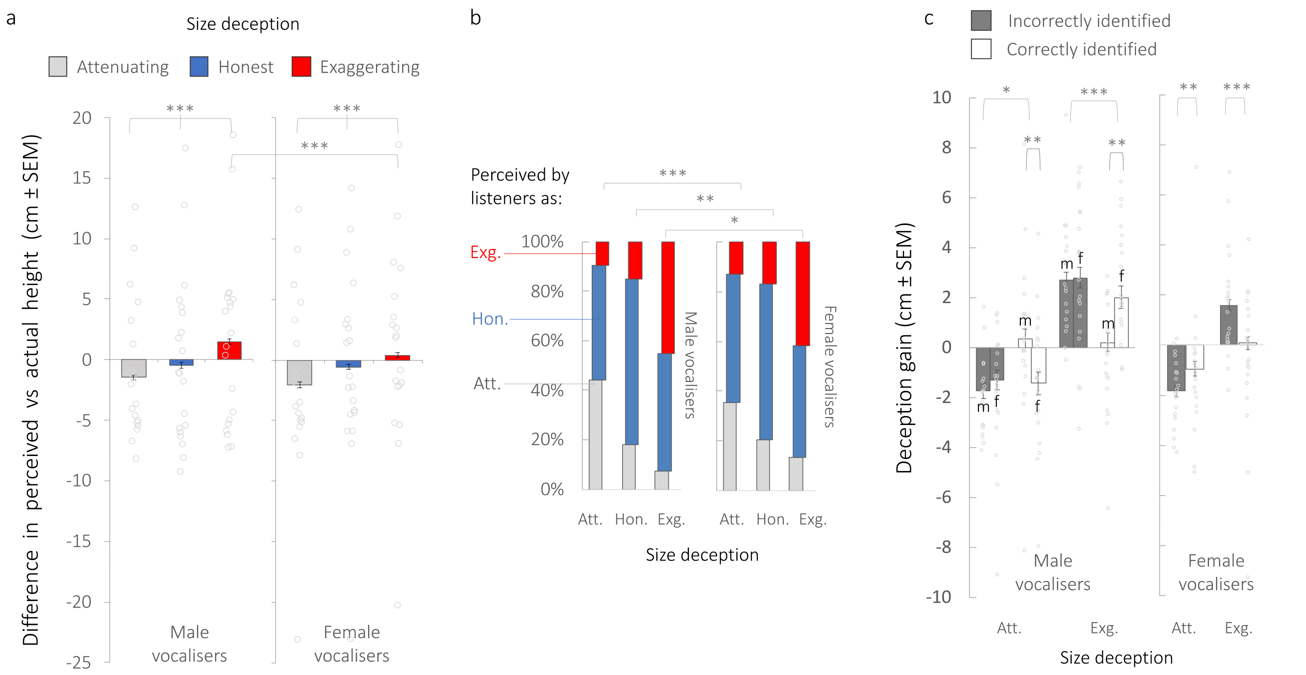

Supplementary Figure 3. Awareness reduces bias: Listeners recalibrate height judgments for signals correctly and concurrently detected as deceptive (Experiment 2) – bar graphs with overlaid dot plots. (a) Bias in height judgments shown as the mean difference (±SEM) between perceived and actual heights of vocalisers, in cm, for honest vocal signals (blue bars) and deceptive vocal signals (attenuating = grey bars, exaggerating = red bars). Estimated marginal means and pairwise comparisons derive from LMMs (see Supplementary Table 11), where all *** p<.001 following Šidák correction for multiple comparisons. Error bars, ±SEM. (b) Percentages of vocalisers that listeners perceived as deceptively exaggerating (red bars) or attenuating (light grey bars) their size, or as producing honest vocal signals (blue bars, center) are shown along the y-axis as a function of intended size deception indicated along the x-axis. Estimated marginal means and pairwise comparisons derive from LMMs (see Supplementary Table 12), where *** p<.001, ** p<.01, * p <.05 following Šidák correction. Tests are two-tailed. (c-d). Bias in listeners' size assessments as a function of whether a listener failed to detect (dark grey bars) or correctly detected (white bars) a vocal signal as deceptive or honest, where panel c shows 'error' in height judgments (mean difference between perceived vs actual heights of vocalisers), and panel d shows 'deception gain' in height judgments (mean difference between perceived height from honest signals and perceived height from deceptive signals). Panel d also illustrates the lower degree of deception gain for male vocalisers (left side) when correctly detected as cheating by other male listeners (labelled with 'm') compared to when detected by female listeners ('f'). Estimated marginal means and pairwise comparisons derive from LMMs (see Supplementary Tables 13 and 14, *** p<.001, ** p<.01, * p<.05 following Šidák correction. Overlaid dot plots show the full distribution across vocalisers, each dot representing a single vocaliser (n=40, 20 males, 20 females), with raw difference scores averaged across listeners (n=98) and within vocalisers of each sex; and in the case of male vocalisers in panel c, also within each listener sex, where n=59 male listeners, 39 female listeners. All tests are two-tailed. Error bars, ±SEM. Acronyms: Att. (attenuating); Hon. (honest); Exg. (exaggerating); m (male listeners); f (female listeners). All data derive from Experiment 2 based on 120 vocal stimuli produced by n=40 vocalisers (20 males, 20 females) in each of three size conditions (honest, attenuating, exaggerating) and judged by n=98 listeners, each of whom rated all 120 vocal stimuli (see Methods). Source data are provided as a Source Data file.

Supplementary References

1. Glasberg, B. R. & Moore, B. C. Derivation of auditory filter shapes from notched-noise data. *Hear. Res.* **47**, 103–138 (1990).
2. Traunmüller, H. *Auditory scales of frequency representation*. (1997). [online tutorial]
