# Supplementary material for: Efficacy in deceptive vocal exaggeration of human body size
Source: Nat Commun. 2021 Feb 12;12:968. doi: 10.1038/s41467-021-21008-7 (PMC7881139; doi:10.1038/s41467-021-21008-7)
Supplement: Supplementary file 3 — Reporting Summary [file 41467_2021_21008_MOESM3_ESM.pdf]

nature research

Corresponding author(s): KATARZYNA PISANSKI

Last updated by author(s): Dec 19 2020

Reporting Summary

Nature Research wishes to improve the reproducibility of the work that we publish. This form provides structure for consistency and transparency in reporting. For further information on Nature Research policies, see our Editorial Policies and the Editorial Policy Checklist.

Statistics

For all statistical analyses, confirm that the following items are present in the figure legend, table legend, main text, or Methods section.

n/a	Confirmed	
☐	☒	The exact sample size (*n*) for each experimental group/condition, given as a discrete number and unit of measurement
☐	☒	A statement on whether measurements were taken from distinct samples or whether the same sample was measured repeatedly
☐	☒	The statistical test(s) used AND whether they are one- or two-sided<br>*Only common tests should be described solely by name; describe more complex techniques in the Methods section.*
☐	☒	A description of all covariates tested
☐	☒	A description of any assumptions or corrections, such as tests of normality and adjustment for multiple comparisons
☐	☒	A full description of the statistical parameters including central tendency (e.g. means) or other basic estimates (e.g. regression coefficient) AND variation (e.g. standard deviation) or associated estimates of uncertainty (e.g. confidence intervals)
☐	☒	For null hypothesis testing, the test statistic (e.g. *F*, *t*, *r*) with confidence intervals, effect sizes, degrees of freedom and *P* value noted<br>*Give P values as exact values whenever suitable.*
☒	☐	For Bayesian analysis, information on the choice of priors and Markov chain Monte Carlo settings
☐	☒	For hierarchical and complex designs, identification of the appropriate level for tests and full reporting of outcomes
☐	☒	Estimates of effect sizes (e.g. Cohen's *d*, Pearson's *r*), indicating how they were calculated

Our web collection on statistics for biologists contains articles on many of the points above.

Software and code

Policy information about availability of computer code

Data collection	Data from psychoacoustic playback experiments were collected using the software Syntoolkit, version 1 (2019-2020), a platform for designing and implementing multi-sensory research experiments developed at the University of Sussex, UK (URL: www.syntoolkit.org). Reference: Hughes, J. E., Gruffydd, E., Simner, J., & Ward, J. (2019). Synaesthetes show advantages in savant skill acquisition: Training calendar calculation in sequence-space synaesthesia. Cortex, 113, 67-82.
Data analysis	Acoustic analyses were performed using PRAAT, a popular open-access acoustic analysis and resynthesis software, version 6.0.21 (URL: www.praat.org). Reference: Boersma, P. & Weenink, D. Praat: Doing phonetics by computer v 6.0.21. (2018). Statistical analyses were performed in SPSS version 24 (IBM).

For manuscripts utilizing custom algorithms or software that are central to the research but not yet described in published literature, software must be made available to editors and reviewers. We strongly encourage code deposition in a community repository (e.g. GitHub). See the Nature Research guidelines for submitting code & software for further information.

Data

Policy information about availability of data

All manuscripts must include a data availability statement. This statement should provide the following information, where applicable:

- Accession codes, unique identifiers, or web links for publicly available datasets
- A list of figures that have associated raw data
- A description of any restrictions on data availability

The authors declare that all data generated or analysed during this study are included in this article and in its supplementary information files, including source data for all figures provided as a Source Data file. Datasets and voice stimuli (n=120 audio WAV files) are also available on the Open Science Framework (https://osf.io/r7gzb/, DOI 10.17605/OSF.IO/R7GZB).

Field-specific reporting

Please select the one below that is the best fit for your research. If you are not sure, read the appropriate sections before making your selection.

☐ Life sciences ☒ Behavioural & social sciences ☐ Ecological, evolutionary & environmental sciences

For a reference copy of the document with all sections, see nature.com/documents/nr-reporting-summary-flat.pdf

Behavioural & social sciences study design

All studies must disclose on these points even when the disclosure is negative.

Study description	This study involves a quantitative experimental design, including spectro-temporal acoustic analysis and psychoacoustic playback experiments.
Research sample	Psychoacoustic experiments include data from 195 adult listeners. Experiment 1, n = 97, 59 participants indicated male gender (aged 18-63, 9.2 sd), 36 indicated female gender (aged 18-63, 11.1 sd) and two indicated their gender as 'other'. Experiment 2, n = 98, 59 participants indicated male gender (aged 21-71, 9.7 sd), and 39 indicated female gender (aged 18-55, 9.4 sd). While all listeners were English speaking to ensure full comprehension of instructions, the sample is representative, with a broad range of ages and a roughly equal sex ratio. Participants were recruited from the general population, thus also representing various socioeconomic backgrounds and educations levels. Sample sizes were determined prior to testing (see Sampling strategy below).<br><br>Vocal stimuli derived from 40 adult English speakers with a representative range of heights and an even sex ratio: 20 men (mean age 19.6 ± 2.4 sd) whose heights ranged from 161 cm to 187 cm (mean height 178.4 ± 7 cm sd) and 20 women (mean age 19.1 ± 1.6 sd) whose heights ranged from 147 cm to 185 cm (mean height 164.9 ± 7.9 sd). These voice stimuli represent a sub-set from a larger sample of vocal stimuli used in a previous study (see Pisanski et al., 2016). The height distributions of vocalisers are representative of the general population; they closely parallel those observed in large cross-cultural samples of adults (men 178 ± 6.58 cm, n=1334; women 165.96 ± 6.64 cm, n=871; see Pisanski et al., 2014).<br><br>References: Pisanski, K. et al. Volitional exaggeration of body size through fundamental and formant frequency modulation in humans. Sci. Rep. 6, 34389 (2016); Pisanski, K. et al. Vocal indicators of body size in men and women: a meta-analysis. Anim. Behav. 95, 89–99 (2014).
Sampling strategy	Participants taking part in psychoacoustic experiments (n=195) were recruited via Amazon's online recruitment platform, Mechanical Turk. Sample sizes were determined prior to testing to achieve a minimum of 50 height judgments per vocal stimulus per experiment for a statistical power of 80%, in order to obtain a small-to-medium effect size in regressions between perceived and actual vocaliser height. While high inter-rated agreement (alphas > 0.80, ps < 0.001) among listeners is typically achieved with relatively small sample sizes (e.g., less than 15 listeners per sex for voice-based judgments of dominance or attractiveness), earlier studies on human vocal communication of body size have generally failed to find significant correlations between perceived and actual height in one or both sexes of vocalisers with samples of fewer than 25 listeners per vocal stimulus (Collins, 2000; Bruckert et al., 2006; van Dommelen & Moxness, 2005).<br><br>References: van Dommelen, W. A. & Moxness, B. H. Acoustic parameters in speaker height and weight identification: sex-specific behaviour. Lang. Speech 38 (Pt 3), 267–287 (1995); Collins, S. A. Men's voices and women's choices. Anim. Behav. 60, 773–780 (2000); Bruckert, L., Lienard, J.-S., Lacroix, A., Kreutzer, M. & Leboucher, G. Women use voice parameters to assess men's characteristics. Proc. R. Soc. B Biol. Sci. 273, 83–89 (2006).
Data collection	Participants taking part in psychoacoustic experiments completed a short demographic questionnaire and completed one of two playback experiments, custom designed in Syntoolkit software, each involving two tasks: judging vocaliser height, and detecting vocal size deception. In Experiment 1, listeners performed these two tasks in separate, consecutive blocks. In each task listeners rated the voices of a random sample of 10 male and 10 female vocalisers, in each of three voice conditions (honest, exaggerated and attenuated size) resulting in 60 trials per task, or a total of 120 trials per listener. Height judgments preceeded the deception detection task so as not to prime nor bias listeners toward contemplating deception when judging height, thus reflecting a more ecologically valid experimental design. Importantly, the same voice stimuli were presented in both tasks within listeners to allow for meaningful comparisons. In Experiment 2, an independent sample of listeners performed the same tasks, however the tasks were now performed concurrently for each vocal stimulus. Thus, listeners first indicated whether or not they perceived a vocaliser as deceptively altering their voice to sound larger or smaller, and then judged the height of that same vocaliser, within the same experimental trial. Listeners judged all 20 male and 20 female vocalisers, in each of three voice conditions (honest, exaggerated and attenuated size), for a total of 120 trials per listener. In both Experiments, listeners were presented with a single vocal stimulus on each trial. Voice stimuli were blocked by sex of vocaliser, and block order and stimulus presentation within each block were randomized. Listeners were instructed to wear headphones and not to adjust their volume settings throughout the experiment; this was verified during debriefing. All experiments were double blind.
Timing	Experiment 1 psychoacoustic playback data were collected in May 2018. Experiment 2 data were collected in July 2020. The second experiment was specifically designed to test the prediction, arising from the results of the first experiment, that priming listeners to seek deception when judging the heights of vocalisers will elicit greater recalibration of height judgments; this was supported by the results.
Data exclusions	Two-hundred participants took part in psychoacoustic playback experiments and data were analysed from 195. In Experiment 1, three participants did not finish the study and were thus excluded from analyses. In Experiment 2, two participants provided random

	responses and were thus excluded from analyses.
Non-participation	No participants dropped out of the study or declined participation.
Randomization	Participants were not allocated into experimental groups, as Experiments 1 and 2 were conducted at separate times. In each experiment, voice stimuli were blocked by sex of vocaliser and the order in which participants judged male or female voices was randomized. Voice stimulus presentation within each block was also randomized.

Reporting for specific materials, systems and methods

We require information from authors about some types of materials, experimental systems and methods used in many studies. Here, indicate whether each material, system or method listed is relevant to your study. If you are not sure if a list item applies to your research, read the appropriate section before selecting a response.

Materials & experimental systems

n/a	Involved in the study
☒	☐ Antibodies
☒	☐ Eukaryotic cell lines
☒	☐ Palaeontology and archaeology
☒	☐ Animals and other organisms
☐	☒ Human research participants
☒	☐ Clinical data
☒	☐ Dual use research of concern

Methods

n/a	Involved in the study
☒	☐ ChIP-seq
☒	☐ Flow cytometry
☒	☐ MRI-based neuroimaging

Human research participants

Policy information about studies involving human research participants

Population characteristics	See above
Recruitment	Participants taking part in psychoacoustic experiments were recruited via the online platform, Amazon Mechanical Turk. All participants provided informed consent and were reimbursed monetarily at the recommended ethical rate of $0.13 USD per minute ($3.50 - $5.15 USD). Participants were randomly selected; the only inclusion criteria were age (18+) and language (English). Data collection was stopped when n=100 participants completed either experiment; this sample size was pre-determined (see Sampling strategy above). Regarding potential self-selection bias, the study was conducted online, hence only participants with access to the internet and a computer could take part in this research, potentially limiting the generalisability of the results to less industrialised nations.
Ethics oversight	This research was approved by the University of Sussex's Life Sciences & Psychology Cluster-based Research Ethics Committee (C-REC; Certificates of approval: ER/KP292/11 and ER/REBY/12) and complies with the American Psychological Association's Ethical Principles of Psychologists and Code of Conduct.

Note that full information on the approval of the study protocol must also be provided in the manuscript.
