# Supplementary material for: Efficacy in deceptive vocal exaggeration of human body size
Source: Nat Commun. 2021 Feb 12;12:968. doi: 10.1038/s41467-021-21008-7 (PMC7881139; doi:10.1038/s41467-021-21008-7)
Supplement: Supplementary file 4 — Description of Additional Supplementary Files [file 41467_2021_21008_MOESM4_ESM.pdf]

Description of Additional Supplementary Files

File name: Supplementary Data 1
Description: This dataset includes all raw data from Experiment 1 (spreadsheet 2) and Experiment 2 (spreadsheet 3) with a corresponding legend (spreadsheet 1).
