# Supplementary material for: Efficacy in deceptive vocal exaggeration of human body size
Source: Nat Commun. 2021 Feb 12;12:968. doi: 10.1038/s41467-021-21008-7 (PMC7881139; doi:10.1038/s41467-021-21008-7)
Supplement: Supplementary file 2 — Peer Review File [file 41467_2021_21008_MOESM2_ESM.pdf]

Reviewer #1 (Remarks to the Author):

This paper reports the result of an acoustic analysis and two perception studies showing that 1) most generally, male and female speakers volitionally lower formant spacing and F0 in spoken vowels to sound larger, and raise these features to sound smaller, 2) listeners' judgments of height are affected by these changes, with male speakers able to affect judgments to a greater extent than females, and 3) correct identification of deceptive vocalizing mitigates the effect, but not completely. These results are interpreted as evidence that deception can be effective while still maintaining honesty on average in relative height assessment across speakers. Overall, the research is meticulous and the work is well described. I think pending some minor revisions the paper should be published in Nature Communications.

The acoustic analysis revealed an interesting sex difference that speaks to possible sexual selection on size exaggeration in men. For the perception experiments, I feel the analysis is sound though I would have preferred to see the judgments analyzed in signal detection terms that would provide a superior quantitative assessment of how biases were altered by the correct recognition of exaggeration attempts, including effects on overall judgment accuracy.

Theoretically, I appreciate the motivation to examine human deception dynamics in an effort to solve issues related to reliability in animal signaling. But I feel some issues, probably most terminological, could be addressed to make the argument clearer. There are some statements that obscure the specific signaling arguments.

Line 41: "animal signals are now predominantly regarded as selfish, functioning largely for the benefit of the signaller by manipulating receivers." This wording "functioning largely for the benefit of the signaler" should be changed because the emphasis on signaler benefits contradicts the ESS logic. Receivers must gain benefits generally for signals to evolve and be maintained. The authors say this later, but this sentence should be altered.

Line 138: "These acoustic analyses indicate that reliable formant-based information signalling inter-individual differences in body size is present in the human voice even during size deception, and thus, that listeners may be able to reliably gauge relative size from deceptive vocal signals." Shouldn't "signaling" be "indicating"? I don't think the authors wish to claim formant structure constitutes an honest signal (i.e., that feature was not selected to affect that judgment). This is separate from the argument that volitional formant adjustment constitutes a potentially deceptive signal.

I think the authors want to argue that 1) formant spacing constitutes an honest index of body size, but is not an evolved signal, 2) F0 is a signal that functions during intrasexual competition (but this is not really specified) and 3) volitional changes in formant spacing and F0 constitute deceptive signals that still maintain some honesty due to various constraints. This line of reasoning seems mostly right to me, but questions remain regarding the function of sexually dimorphic pitch which is not related to intrasexual body size. The question of pitch should be better addressed especially given recent debates in the literature regarding its various effects and evolution.

This brings us to the question of formidability. This work looks exclusively at height, which the authors briefly state is a proxy for formidability. To what extent is the arms race, and the evolved psychology, geared towards signaling and assessing formidability rather than height? If so, then the dynamics described here related only to height might be misleading in ways. I think it is important for the authors to lay out specifically where they stand on vocal signals of formidability. Are they arguing that formant spacing itself is a cue of height, but purposeful demonstrations that emphasize that spacing constitute a signal of formidability? And if so, what is the justification for considering that behavior as honest? Is it possible there is no deception at all? If relative size is maintained, and there are hard limits on what is possible, and exaggerated efforts are often recognizable, to what extent is the concept of deception warranted? In situations of conflict, do speakers actually produce vocalizations

that often sound like a person trying to sound taller?

This relates to a final point regarding the stimuli and the pattern of deception detection and subsequent judgments. Without hearing the stimuli, I have a few concerns. Using vowels afford proper formant measurement, but sacrifice ecological validity as realistic tokens of communicative vocal behavior. I suspect that many of these vocalizations sound overacted, and thus could induce task demands that could obscure actual deception (in both experiments). In other words, listeners hear a silly attempt at sounding large, and they correctly identify the attempt (Exp 2), and then are lead in the task unconsciously to slightly overestimate the height of the speaker (or underestimate of course). Put simply, is it possible these results are just a byproduct of the experimental paradigm? I'd be interested in how the authors address this concern.

Reviewer #2 (Remarks to the Author):

This works investigates the ability of human listeners to detect body size (height) differences in other humans using sexually dimorphic vocal utterances. The work provides what I thought was a story-form depiction and a rare test of whether deception can be identified through utterances, and whether correctly or incorrectly identified calls carry any fitness benefits. I thought the paper's findings provide evidence for a hard-to-test phenomenon within the animal communication literature and is applicable to a rather large audience interested in vocal communication, adaptive sexual dimorphisms, evolutionary arms races, and signal / cheater detection.

In general, the author's interpretation of the results are supported by the data. I especially liked the follow-up experiment (exp2) to further investigate how priming the deception task impacted rater's responses. While there was no major limitation to the data or methodology, I do have one question regarding a statistical technique used, and another question regarding further analyses of sex differences. I believe the authors will be able to address these points and other concerns outlined below for this paper to make an important contribution to their field.

Why Spearman's rho for conducting the (for example) perceived / actual height relationships and not an attempt to make the distributions more normal (various transformations)? You indicate that the outliers in the data didn't significantly impact the statistical trends. How different is the data when using Pearson's r? To my knowledge rho is used when you want to boil down data into rank-ordered units.

Line 276 – "In addition, our finding that listeners can effectively gauge the relative heights of deceivers predicts an asymmetry in costs for male and female listeners…….This sexual asymmetry supports the hypothesis that male-male competition is the primary mechanism of selection on men's sexually dimorphic traits…" How does your data specifically speak to this? This seems like it needs to be cleared up.

Not disconnected from above, you considered the sex of the listener as a variable in some of the analyses, but didn't find an effect in earlier models. I didn't see this variable later on, for example in the 'primed to detect deception' paradigm. Was sex of listener not considered here? Would differences, or the lack thereof speak to the male-male competition hypothesis (e.g., were males more accurate in detecting height when correctly identifying exaggeration compared to females? Or just more accurate in identifying exaggeration in other males compared to females?

About line 50 – briefly explain what honest signaling is to readers. You jump into "honest on average," but explaining the costs incurred from honest signaling (and its relation to handicap principle) is warranted.

Figure 3a. - can the bars showing exaggeration, honesty, and attenuation be all put on the X axis? This will allow the reader to judge the percentages more accurately.

About Line 110 – can the authors mention the utterances used in this study? I understand this is more for the methods section but it would give the reader some idea of the task at hand. Same thing for experiment discussed at line 143.

Line 88 – you may want to explain to readers what formant spacing is (or state that it's shown in graphical form in fig. 1)?

Supplementary tables, provide sample sizes for all studies

It's interesting to see that in fig 2, there continues to be a positive relationship between actual and perceived height even in the attenuation condition for females. Is this from the constraints in modulating their vocal tract?

Reviewer #3 (Remarks to the Author):

Review Pisanski & Reby "Efficacy in deceptive vocal exaggeration of human body size"

This is an interesting and convincing study of a phenomenon – deceptive vocal signals – of long-standing interest in the evolution of communication. The authors use humans as study subjects because they can easily be directed to modify their voices – a clever way around otherwise intractable problems that arise with most other animal species. They find both that deception is effective (it in fact deceives listeners) but also that listeners are somewhat aware of deception, and when they are aware can discount its effects. This is consistent with evolutionary models of an arm's race between signalers and perceivers, and is really the first time such direct evidence has been obtained.

The study is really well done. I have only one, relatively minor, methodological issue. Regarding the finding that men (and particularly tall men) shifted more than women or short men- could this be an artefact of starting size? Because a long vocal tract will change more (in cm, and thus in Hz) if it lengthens by 10% than will a short vocal tract, we might predict this effect on purely physical grounds.

I'd like to see a calculation of %age change in estimated VTL to see if this finding is really about effectiveness of deception, rather than just a byproduct of vocal tract starting dimensions.

Minor changes:
The recommendation in the voice community is to use a lowercase "f" for fundamental frequency, and uppercase "F" for formant frequencies, to avoid confusion among these fundamentally different frequency measures. So I recommend changing F0 -> f0 throughout.

Titze, I. R., Baken, R. J., Bozeman, K. W., Granqvist, S., Henrich, N., Herbst, C. T., Howard, D. M., Hunter, E. J., Kaelin, D., Kent, R. D., Kreiman, J., Kob, M., Löfqvist, A., McCoy, S., Miller, D. G., Noé, H., Scherer, R. C., Smith, J. R., Story, B. H., Svec, J. G., Ternström, S., and Wolfe, J. (2015). "Toward a consensus on symbolic notation of harmonics, resonances, and formants in vocalization " J. Acoustic. Soc. Am. 137, 3005.

Anatomical differences between men and women are referred to but not properly references. The authors should give at least one reference to back this up, e.g.: Fitch, W. T., and Giedd, J. (1999). "Morphology and development of the human vocal tract: a study using magnetic resonance imaging,"

J. Acoustic. Soc. Am. 106, 1511-1522.

Additions or changes to text:
animal signals AMONG UNRELATED INDIVIDUALS are now predominantly regarded as selfish

abnormally low formant frequency spacing (ΔF) GIVEN the animal's true size

maximally exploiting deep-SEATED sound-size correspondences

nevertheless failed to detect DECEPTION approximately half of the time

formability
-> formidability

REVIEWER COMMENTS

Reviewer #1 (Remarks to the Author):

This paper reports the result of an acoustic analysis and two perception studies showing that 1) most generally, male and female speakers volitionally lower formant spacing and F0 in spoken vowels to sound larger, and raise these features to sound smaller, 2) listeners' judgments of height are affected by these changes, with male speakers able to affect judgments to a greater extent than females, and 3) correct identification of deceptive vocalizing mitigates the effect, but not completely. These results are interpreted as evidence that deception can be effective while still maintaining honesty on average in relative height assessment across speakers. Overall, the research is meticulous and the work is well described. I think pending some minor revisions the paper should be published in Nature Communications.

We thank the reviewer for their positive remarks and for their encouragement. Below we respond to each specific comment.

The acoustic analysis revealed an interesting sex difference that speaks to possible sexual selection on size exaggeration in men. For the perception experiments, I feel the analysis is sound though I would have preferred to see the judgments analyzed in signal detection terms that would provide a superior quantitative assessment of how biases were altered by the correct recognition of exaggeration attempts, including effects on overall judgment accuracy.

We agree that a signal detection analysis would have been interesting particularly in highlighting false positives. However, we are confident that our analyses provide conclusive and replicable results that are adequate for answering our hypotheses and that are sufficiently robust to support our interpretations.

Theoretically, I appreciate the motivation to examine human deception dynamics in an effort to solve issues related to reliability in animal signaling. But I feel some issues, probably most terminological, could be addressed to make the argument clearer. There are some statements that obscure the specific signaling arguments.

Line 41: "animal signals are now predominantly regarded as selfish, functioning largely for the benefit of the signaller by manipulating receivers." This wording "functioning largely for the benefit of the signaler" should be changed because the emphasis on signaler benefits contradicts the ESS logic. Receivers must gain benefits generally for signals to evolve and be maintained. The authors say this later, but this sentence should be altered.

We thank the reviewer for this helpful comment. In response to this and to a comment by reviewer 3 on the same sentence, we have changed the text to:

> **"While earlier theories saw animal communication as a cooperative exchange of information[19], the production of animal signals during communication between unrelated individuals is now predominantly regarded as a selfish behaviour[2,19], whereby signallers attempt to manipulate the responses of receivers." (lines 41-44)**

As in the original text, subsequent sentences then describe the potential benefits for receivers and selection pressure on both signalers and receivers to maximise their own fitness benefits.

Line 138: "These acoustic analyses show that reliable formant-based information signalling inter-individual differences in body size is present in the human voice even during size deception, and thus, that listeners may be able to reliably gauge relative size from deceptive vocal signals." Shouldn't "signaling" be "indicating"? I don't think the authors wish to claim formant structure constitutes an honest signal (i.e., that feature was not selected to affect that judgment). This is separate from the argument that volitional formant adjustment constitutes a potentially deceptive signal.

Here too we agree with the reviewer and have changed "signalling" to "indicating".

I think the authors want to argue that 1) formant spacing constitutes an honest index of body size, but is not an evolved signal, 2) F0 is a signal that functions during intrasexual competition (but this is not really specified) and 3) volitional changes in formant spacing and F0 constitute deceptive signals that still maintain some honesty due to various constraints. This line of reasoning seems mostly right to me, but questions remain regarding the function of sexually dimorphic pitch which is not related to intrasexual body size. The question of pitch should be better addressed especially given recent debates in the literature regarding its various effects and evolution.

This is an important point and we have now integrated the role of voice pitch in size communication more extensively into the introductory text (Page 4). The paragraph now reads:

> **"Humans also possess a descended and sexually dimorphic larynx, with men boasting longer vocal tracts (reduced Δ*F*) and longer vocal folds (lower fundamental frequency or pitch, *f*0) than women[13,34,35] (Fig. 1). Although Δ*F* scales allometrically with vocal tract length and thus predicts body size, both between and within adult sexes, *f*0 is a poor predictor of human height at the intrasexual level[13,35,36] (see Fig. 1). Yet, despite strongly associating not only Δ*F* but also *f*0 with physical largeness[15,16,37], listeners can gauge relative body size from modal speech and nonverbal vocalisations[15,37,38]. Critically, however, while we have recently shown that men and women can behaviourally lower their voice Δ*F* and *f*0 to further exaggerate their body size, and strength[39,40], remarkably little is known about the role of such deception in size communication." (lines 93-101)**

We also refer the reviewer to Fig. 1 where we further discuss the anatomical mechanisms and relative roles of f0 and formants in size communication.

This brings us to the question of formidability. This work looks exclusively at height, which the authors briefly state is a proxy for formidability. To what extent is the arms race, and the evolved psychology, geared towards signaling and assessing formidability rather than height? If so, then the dynamics described here related only to height might be misleading in ways. I think it is important for the authors to lay out specifically where they stand on vocal signals of formidability. Are they arguing that formant spacing itself is a cue of height,

but purposeful demonstrations that emphasize that spacing constitute a signal of formidability?

Our experiment was specifically designed to assess the effect of deception on the vocal communication of body size. We chose this trait because body size is a key predictor of fitness in numerous species, many of which show anatomical or behavioural adaptations for vocal size exaggeration, and thus an important target for sexual selection and deceptive signalling. Moreover, body size is known to be encoded in formant spacing in many animals, including humans, and *actual size* and *perceived size* are quantifiable metrics that can be measured objectively and that allow us to directly assess the reliability of size communication (and deception) in vocal communication. While body size clearly contributes to one's perceived formidability, we agree that our results are specific to size perception and do not systematically generalize to the perception of formidability, and that, as such, should be more cautiously formulated. In order to avoid any potential confusion, we have thus decided to:

- **delete "and formidability" (line 99)**
- **change lines 169-170 to read, "consistent with previous suggestions that the exaggeration of apparent *body size* is under stronger sexual selection in male than female vocal signals [10, 14-16]…**
- **replace "formidability" by "traits" (line 324).**

As before we also note in our discussion that further research is needed to elucidate the production and, in particular, the perception, of deceptive vocal signals of dominance, strength, attractiveness, and other biologically and socially relevant traits. (Page 13)

And if so, what is the justification for considering that behavior as honest? Is it possible there is no deception at all? If relative size is maintained, and there are hard limits on what is possible, and exaggerated efforts are often recognizable, to what extent is the concept of deception warranted?

Our data clearly show that exaggerated efforts are not always recognizable (deceit is correctly detected only half of the time, see Figs. 2a and 4b), and when this is the case, listeners' absolute size judgments are significantly overestimated (for size exaggeration) and underestimated (for size attenuation). Moreover, even when deceptive signals are detected, they can remain effective. As such we consider that we are well justified to interpret our observation as attempts to deceive, that are often successful. With regards to the qualification of "honesty", this refers to the fact that while absolute height is inflated by size exaggeration, relative speaker height (that is, interindividual differences in speaker heights) are broadly maintained: taller men exaggerating still sound taller than shorter men exaggerating. These observations are consistent with the concept of "honesty in an exaggerated signal".

In situations of conflict, do speakers actually produce vocalizations that often sound like a person trying to sound taller?

We do not report data on actual conflicts as this was not the aim of this specific study, but again, our data suggest that size exaggerators are often not detected as deceptive, or that receivers do not detect the full extent of their exaggeration. As such it is unlikely that deceivers systematically "sound like a person trying to sound taller". And, even if this

were the case, our results show that deception can remain efficient, even when it is detectable. Below we elaborate further regarding the reviewers comment regarding task demands and invite the reviewer to listener to the supplementary audio files.

This relates to a final point regarding the stimuli and the pattern of deception detection and subsequent judgments. Without hearing the stimuli, I have a few concerns. Using vowels afford proper formant measurement, but sacrifice ecological validity as realistic tokens of communicative vocal behavior.

We agree that using vowels introduces a trade-off with ecological validity however we felt that standardizing our stimuli was paramount because, as the reviewer acknowledges, this ensures the reliability of our acoustic analyses, and also reduces the noise that would have inevitably resulted from the use of e.g. longer verbal stimuli. In our discussion we fully acknowledge that "Research into vocal deception of a wide range of traits[48] and states[47], particularly in multi-modal real-world contexts[49], is needed to further elucidate the functions and tangible consequences of deceit in complex social environments." (lines 327-329)

I suspect that many of these vocalizations sound overacted, and thus could induce task demands that could obscure actual deception (in both experiments). In other words, listeners hear a silly attempt at sounding large, and they correctly identify the attempt (Exp 2), and then are lead in the task unconsciously to slightly overestimate the height of the speaker (or underestimate of course). Put simply, is it possible these results are just a byproduct of the experimental paradigm? I'd be interested in how the authors address this concern.

We thank the reviewer for these interesting remarks and reassure the reviewer that our stimuli did not sound overacted or like silly attempts to exaggerate one's size. We have included all 120 voice stimuli in supplemental materials and also uploaded the WAV files to the Open Science Framework (https://osf.io/r7gzb/, DOI 10.17605/OSF.IO/R7GZB) so that the reviewer can confirm this for themselves. A link to the online stimuli has also been included in our Methods for readers. In particular we draw the reviewer's attention to several voice stimuli that were frequently detected as deceitful (i.e., more than 75% correct detection) but do not sound at all silly e.g., males: m_5323_exaggerating, m_5428_exaggerating, m_5310_attenuating; m_5503_attenuating; females: f_5254_exaggerating, f_5426_exaggerating, f_5508_attenuating. We also note a random selection of examples of deceptive voice stimuli that often went undetected (i.e., less than 25% correct detection) e.g., males: m_5303_exaggerating, m_5410_exaggerating, m_5409_attenuating; m_5429_attenuating; females: f_5320_exaggerating, f_5338_exaggerating, f_5424_attenuating, f_5426_attenuating.

It's important to also underscore that speakers were asked to "reproduce the vowels while sounding physically large", and again while "sounding physically small" but not to sound as large or small as possible, thus reducing the probability of overacted portrayals. Moreover, as mentioned above, in Experiment 1, deception was not always detectable (e.g. size exaggeration by male vocalisers went undetected in 49% of cases) and remained largely effective even when it was.

Reviewer #2 (Remarks to the Author):

This works investigates the ability of human listeners to detect body size (height) differences in other humans using sexually dimorphic vocal utterances. The work provides what I thought was a story-form depiction and a rare test of whether deception can be identified through utterances, and whether correctly or incorrectly identified calls carry any fitness benefits. I thought the paper's findings provide evidence for a hard-to-test phenomenon within the animal communication literature and is applicable to a rather large audience interested in vocal communication, adaptive sexual dimorphisms, evolutionary arms races, and signal / cheater detection.

In general, the author's interpretation of the results are supported by the data. I especially liked the follow-up experiment (exp2) to further investigate how priming the deception task impacted rater's responses. While there was no major limitation to the data or methodology, I do have one question regarding a statistical technique used, and another question regarding further analyses of sex differences. I believe the authors will be able to address these points and other concerns outlined below for this paper to make an important contribution to their field.

We thank the reviewer for their positive and insightful comments, and provide detailed responses to their comments and suggestions below.

Why Spearman's rho for conducting the (for example) perceived / actual height relationships and not an attempt to make the distributions more normal (various transformations)? You indicate that the outliers in the data didn't significantly impact the statistical trends. How different is the data when using Pearson's r? To my knowledge rho is used when you want to boil down data into rank-ordered units.

We used a nonparametric correlation coefficient to assess bivariate relationships where the relationships could be either linear or monotonic, and where variables were in some cases non-normally distributed, as Pearson's *r* is limited to linear relationships between normally distributed variables. We thus used Spearman's rho to examine bivariate relationships between voice frequency shifts and vocaliser height (Table S3), absolute voice frequencies and vocaliser height (Table S4), and between voice frequencies and perceived vocaliser height in Experiment 1 (Table S6). However for transparency and comparative purposes we have now re-run these bivariate tests using Pearson's *r* and report these results along with Spearman's *rho* coefficients in Tables S3, S4 and S6. The reviewer will note that the two statistics are highly comparable. Indeed as the reviewer notes, and as stated in our Methods, removing or retaining outliers (there were very few) did not affect the direction of relationships or general statistical trends.

Line 276 – "In addition, our finding that listeners can effectively gauge the relative heights of deceivers predicts an asymmetry in costs for male and female listeners…….This sexual asymmetry supports the hypothesis that male-male competition is the primary mechanism of selection on men's sexually dimorphic traits…" How does your data specifically speak to this? This seems like it needs to be cleared up.

We agree with the reviewer that this paragraph needed clarifying. We have thus edited it to read:

> **"…our finding that listeners can effectively gauge the *relative* heights of deceivers predicts an asymmetry in the impact of deception on male and female listeners[6]. Indeed, assuming all males exaggerate, females should retain the ability to rank *relative* male quality, which is crucial in mate choice. In contrast, males may overestimate the *absolute* size of exaggerating competitors whose deception goes undetected, where the size and strength of a rival compared to oneself is critical[38], and may thus overvalue the cost of continued conflict." (lines 302-307)**

We thank the reviewer for also suggesting additional investigations into listener sex effects, which as described just below, provide additional support for the male-male competition hypothesis.

Not disconnected from above, you considered the sex of the listener as a variable in some of the analyses, but didn't find an effect in earlier models. I didn't see this variable later on, for example in the 'primed to detect deception' paradigm. Was sex of listener not considered here? Would differences, or the lack thereof speak to the male-male competition hypothesis (e.g., were males more accurate in detecting height when correctly identifying exaggeration compared to females? Or just more accurate in identifying exaggeration in other males compared to females?

We are very grateful to the reviewer for this excellent remark. Indeed, we did not find significant main or interaction effects of listener sex in Experiment 1, and thus did not include listener sex in our Experiment 2 models ('primed to detect deception' paradigm). Following the reviewer's suggestion, we have now re-run all LMMs with listener sex (see Supplementary Tables S11, S12, S13, S14) and, as the reviewer predicted, found that male listeners were less biased by size deception in other males when correctly identifying exaggerators (and attenuators) compared to female listeners. We have incorporated this result into Fig 4 (panel d).

We feel that this finding further supports the prediction that that pressure on listeners to counteract deception by recalibrating size judgments for deceptive signals may be maximised in the context of male-male competition, as we now note in the discussion. We also suggest that the specificity of this effect to the second experiment in which listeners were primed to seek deception suggests that male listeners male listeners are particularly attuned to the deceptive signals of other men when an explicit competitive context is induced (Page 12).

About line 50 – briefly explain what honest signaling is to readers. You jump into "honest on average," but explaining the costs incurred from honest signaling (and its relation to handicap principle) is warranted.

We provide a detailed explanation of honest signalling, and of the constraints and costs enforcing it along with several key references for additional reading, and we trust that this is sufficient given the limited space available:

> **"Signal reliability can be imposed by a number of mechanisms including anatomical or physiological constraints (e.g., by-product information[23] or honest *indices*[17]), developmental or metabolic costs[1,24], and reputation or retaliation costs[9,25].**

Constraints and costs, if high enough, can enforce signal honesty." (lines 57-60)

Figure 3a. - can the bars showing exaggeration, honesty, and attenuation be all put on the X axis? This will allow the reader to judge the percentages more accurately.

Below we present figure 3a in the format suggested by the reviewer (left). While we also intuitively felt that this suggested format could be more readable, upon creating this graph, we feel that the original stacked column graph (right) is in fact more easily interpretable, particularly because each column sums to 100%. We trust the reviewer and editor will agree but are happy to use the reviewers suggested format if preferred.

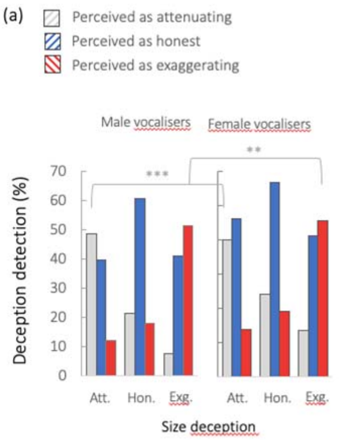

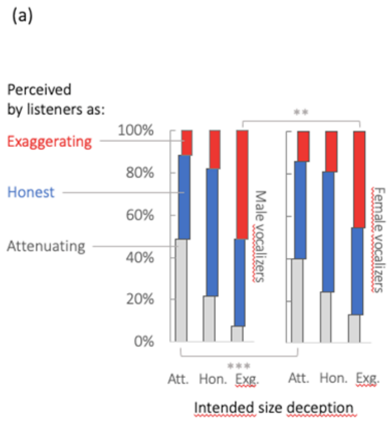

About Line 110 – can the authors mention the utterances used in this study? I understand this is more for the methods section but it would give the reader some idea of the task at hand. Same thing for experiment discussed at line 143.

We have now noted the utterance type (vowels, /α/, /i/, /ε/, /o/, /u/) in the main text.

Line 88 – you may want to explain to readers what formant spacing is (or state that it's shown in graphical form in fig. 1)?

We have edited the text in brackets, which now reads: "(Δ*F*, the overall spacing between any two consecutive formants in the frequency domain, see figure 1) (line 90)

Supplementary tables, provide sample sizes for all studies

We have now provided sample sizes in the footnotes of all supplementary tables.

It's interesting to see that in fig 2, there continues to be a positive relationship between actual and perceived height even in the attenuation condition for females. Is this from the constraints in modulating their vocal tract?

Indeed, there is an interesting sex difference here. The fact that the positive relationship is maintain during size exaggeration in men but during size attenuation in women may derive from an interplay between anatomical and behavioural constraints linked to the

behavioural expression of gender in the normal voice men speaking with an already behaviourally extended vocal tract (e.g. more rounded lips) and women speaking with a behaviorally shortened vocal tract (e.g. more smiley), and thus closer to anatomical limits that are more readily reached during size exaggeration in men and size attenuation in women, reinforcing the correlation. While this interpretation is indeed plausible, we believe that more research (particularly using dynamic MRI) would be needed to substantiate it.

Reviewer #3 (Remarks to the Author):

Review Pisanski & Reby "Efficacy in deceptive vocal exaggeration of human body size"

This is an interesting and convincing study of a phenomenon – deceptive vocal signals – of long-standing interest in the evolution of communication. The authors use humans as study subjects because they can easily be directed to modify their voices – a clever way around otherwise intractable problems that arise with most other animal species. They find both that deception is effective (it in fact deceives listeners) but also that listeners are somewhat aware of deception, and when they are aware can discount its effects. This is consistent with evolutionary models of an arm's race between signalers and perceivers, and is really the first time such direct evidence has been obtained.

We thank the reviewer for their very positive comments.

The study is really well done. I have only one, relatively minor, methodological issue. Regarding the finding that men (and particularly tall men) shifted more than women or short men- could this be an artefact of starting size? Because a long vocal tract will change more (in cm, and thus in Hz) if it lengthens by 10% than will a short vocal tract, we might predict this effect on purely physical grounds. I'd like to see a calculation of %age change in estimated VTL to see if this finding is really about effectiveness of deception, rather than just a byproduct of vocal tract starting dimensions.

We can confirm that these effects are not a byproduct of baseline vocal tract length, as we now show in supplementary Table S1, where we present % change in estimated VTL along with absolute values for honest, attenuated and exaggerated vocal signals. The reviewer will note that in the size exaggeration condition, men increased their apparent vocal tract length from baseline about two and half times more than did women (7.1% in men versus 3% in women). We also report % change for mean Δ*F* and mean *f*0. Here too, even on the Bark scale, men lowered their formant spacing four times more than did women to exaggerate their size.

Minor changes:
The recommendation in the voice community is to use a lowercase "f" for fundamental frequency, and uppercase "F" for formant frequencies, to avoid confusion among these fundamentally different frequency measures. So I recommend changing F0 -> f0 throughout.

Titze, I. R., Baken, R. J., Bozeman, K. W., Granqvist, S., Henrich, N., Herbst, C. T., Howard, D.

M., Hunter, E. J., Kaelin, D., Kent, R. D., Kreiman, J., Kob, M., Löfqvist, A., McCoy, S., Miller, D. G., Noé, H., Scherer, R. C., Smith, J. R., Story, B. H., Svec, J. G., Ternström, S., and Wolfe, J. (2015). "Toward a consensus on symbolic notation of harmonics, resonances, and formants in vocalization " J. Acoustic. Soc. Am. 137, 3005.

We have changed *F*0 to *f*0 throughout the manuscript and supplementary materials, including tables and figures.

Anatomical differences between men and women are referred to but not properly references. The authors should give at least one reference to back this up, e.g.: Fitch, W. T., and Giedd, J. (1999). "Morphology and development of the human vocal tract: a study using magnetic resonance imaging," J. Acoustic. Soc. Am. 106, 1511-1522.

Thank you for the suggestion. We have added this reference to support claims of sexual size dimorphism in human VTL (introductory text and figure 1).

Additions or changes to text:
animal signals AMONG UNRELATED INDIVIDUALS are now predominantly regarded as selfish

Thank you for this helpful comment. In response to this and to a comment by reviewer 1 on the same sentence, we have changed the text to:

> **"While earlier theories saw animal communication as a cooperative exchange of information[19], the production of animal signals in communication between unrelated individuals is now predominantly regarded as a selfish behaviour[2,19], whereby signallers attempt to manipulate the behaviour of receivers." (lines 41-44).**

abnormally low formant frequency spacing (ΔF) GIVEN the animal's true size

maximally exploiting deep-SEATED sound-size correspondences

nevertheless failed to detect DECEPTION approximately half of the time

formability -> formidability

We thank the reviewer for a keen eye and have incorporated these changes.

Reviewer #1 (Remarks to the Author):

The authors have responded well to my issues and I recommend publishing the paper. I listened to the stimuli and I was pleasantly surprised that in fact they did not sound overacted.

Regarding the notation issue raised by R3 (JASA paper by Titze et al.), it is still off. The 0 should be letter o (as in oscillator) not zero. So it's lowercase italicized f, and small letter o.

Congrats on a fine paper!

Reviewer #2 (Remarks to the Author):

The authors have addressed all of my concerns in their revisions. Thank you for showing me what Figure 3a looks like as per my suggestion, and I agree that it didn't make much of a difference in the end, so please continue to use your original format. Thank you for analyzing sex differences in the later models, I'm glad it further supports your framework in the paper. I believe this paper is ready for publication, congratulations!

Reviewer #3 (Remarks to the Author):

The authors have addressed my comments fully. I recommend acceptance.
Tecumseh Fitch

REVIEWERS' COMMENTS
Author responses in bold

Reviewer #1 (Remarks to the Author):

The authors have responded well to my issues and I recommend publishing the paper. I listened to the stimuli and I was pleasantly surprised that in fact they did not sound overacted.

Regarding the notation issue raised by R3 (JASA paper by Titze et al.), it is still off. The 0 should be letter o (as in oscillator) not zero. So it's lowercase italicized f, and small letter o.

Congrats on a fine paper!

We changed all instances of *f*0 to *f*o throughout the manuscript, tables, figures and supplementary materials. In line with the same recommendations by Titze et al, we have also revised all instances of *F*n (formants) to F_n. Thank you so much for your time and invaluable comments.

Reviewer #2 (Remarks to the Author):

The authors have addressed all of my concerns in their revisions. Thank you for showing me what Figure 3a looks like as per my suggestion, and I agree that it didn't make much of a difference in the end, so please continue to use your original format. Thank you for analyzing sex differences in the later models, I'm glad it further supports your framework in the paper. I believe this paper is ready for publication, congratulations!

We are very grateful for your excellent suggestions, particularly including listener sex in the models of the second experiment which has taken our paper to another level. Thank you.

Reviewer #3 (Remarks to the Author):

The authors have addressed my comments fully. I recommend acceptance.
Tecumseh Fitch

Thank you sincerely for your positive feedback, and most of all for your invaluable input and insight.
